# Lipolysis and gestational diabetes mellitus onset: a case-cohort genome-wide association study in Chinese

**DOI:** 10.1186/s12967-023-03902-4

**Published:** 2023-01-25

**Authors:** Miao Zhang, Qing Li, Kai-Lin Wang, Yao Dong, Yu-Tong Mu, Yan-Min Cao, Jin Liu, Zi-Heng Li, Hui-Lu Cui, Hai-Yan Liu, An-Qun Hu, Ying-Jie Zheng

**Affiliations:** 1 Department of Epidemiology, School of Public Health, Fudan University, Shanghai, 200032 China; 2 Key Laboratory for Health Technology Assessment, National Commission of Health and Family Planning, Fudan University, Shanghai, 200032 China; 3 Key Laboratory of Public Health Safety, Ministry of Education, Fudan University, Shanghai, 200032 China; 4 Department of Obstetrics and Gynecology, Anqing Municipal Hospital, Anqing, 246003 China; 5 Department of Clinical Laboratory, Anqing Municipal Hospital, Anqing, 246003 China

**Keywords:** Gestational diabetes mellitus, Genome-wide association study, Metabolism, Lipolysis

## Abstract

**Background:**

Genetic knowledge of gestational diabetes mellitus (GDM) in Chinese women is quite limited. This study aimed to identify the risk factors and mechanism of GDM at the genetic level in a Chinese population.

**Methods:**

We conducted a genome-wide association study (GWAS) based on single nucleotide polymorphism (SNP) array genotyping (ASA-CHIA Bead chip, Illumina) and a case-cohort study design. Variants including SNPs, copy number variants (CNVs), and insertions-deletions (InDels) were called from genotyping data. A total of 2232 pregnant women were enrolled in their first/second trimester between February 2018 and December 2020 from Anqing Municipal Hospital in Anhui Province, China. The GWAS included 193 GDM patients and 819 subjects without a diabetes diagnosis, and risk ratios (RRs) and their 95% confidence intervals (CIs) were estimated by a regression-based method conditional on the population structure. The calling and quality control of genotyping data were performed following published guidelines. CNVs were merged into CNV regions (CNVR) to simplify analyses. To interpret the GWAS results, gene mapping and overexpression analyses (ORAs) were further performed to prioritize the candidate genes and related biological mechanisms.

**Results:**

We identified 14 CNVRs (false discovery rate corrected P values < 0.05) and two suggestively significant SNPs (P value < 0.00001) associated with GDM, and a total of 19 candidate genes were mapped. Ten genes were significantly enriched in gene sets related to lipase (triglyceride lipase and lipoprotein lipase) activity (LIPF, LIPK, LIPN, and LIPJ genes), oxidoreductase activity (TPH1 and TPH2 genes), and cellular components beta-catenin destruction complex (APC and GSK3B genes), Wnt signalosome (APC and GSK3B genes), and lateral element in the Gene Ontology resource (BRCA1 and SYCP2 genes) by two ORA methods (adjusted P values < 0.05).

**Conclusions:**

Genes related to lipolysis, redox reaction, and proliferation of islet β-cells are associated with GDM in Chinese women. Energy metabolism, particularly lipolysis, may play an important role in GDM aetiology and pathology, which needs further molecular studies to verify.

**Supplementary Information:**

The online version contains supplementary material available at 10.1186/s12967-023-03902-4.

## Background

Gestational diabetes mellitus (GDM), defined as abnormally high blood glucose levels among pregnant women without a diabetes diagnosis before pregnancy, is a common complication in pregnancy, and its incidence is increasing worldwide [1, 2]. GDM is associated with macrosomia, which is usually accompanied by dystocia, birth trauma, and caesarean section [3]. GDM patients have higher risks of developing type 2 diabetes mellitus (T2DM), cardiovascular diseases (CVD), and metabolic syndrome (MS) after delivery [4–11], and their offspring also have increased risks for obesity, CVD, diabetes, and MS [8, 12–15].

It is suggested that approximately 80% of GDM cases are related to insulin resistance (IR) [16]. Other hypotheses involving inflammation, oxidative stress, and adipose tissue/endothelial cell dysfunction have also been proposed [17]. However, the pathogenesis of GDM has not been fully clarified [6]. Many risk factors for GDM, including demography, family/fertility history behaviours in pregnancy, and genetic variants, have been identified [12, 16, 18, 19]; however, confounding bias is a problem affecting most studies because of the unclear onset time of GDM. Most genetic studies were candidate gene studies based on T2DM due to the potential common genetic background between the two conditions [20–23], and many shared genes, such as TCF7L2, GCK, MTNR1B, and PPARγ genes, have been found [24, 25]. However, dysglycaemia triggered by pregnancy could have specific mechanisms. Some GDM-associated variants, such as the HKDC1, MTNR1A, ACE, and VDR genes, have not been reported in T2DM genetic studies [24]. To our knowledge, only two genome-wide association studies (GWASs) of GDM have been reported. One study observed 468 GDM cases and 1242 nondiabetic controls in Korea [26]. This study’s controls were women aged ≥ 50 years with unknown GDM histories, which may introduce misclassification bias. The other was conducted in a smaller Chinese sample (103 GDM cases, 115 nondiabetic controls) [27], and no overlapping markers and genes were identified with the previous GWAS.

Screening for GDM based on risk factors, including obesity, first-degree relatives with diabetes, history of GDM or adverse pregnancy outcomes, and glycosuria, will miss approximately one-half of cases [12, 28]. Furthermore, there is no useful predictive model for GDM [29]. The diagnosis of GDM is based on a time-consuming and daunting oral glucose tolerance test (OGTT). Preventive actions are still contentious and ineffective [6, 29–33]. The available management strategy for GDM is limited if only lifestyle changes and the use of insulin or oral antidiabetics are considered [31–33]. In summary, further studies of the pathogenesis of GDM are imperative to put forwards innovative precocious preventive or therapeutic interventions, and GWASs could help find clues from the whole genome level with few confounders and without a hypothesis.

The average incidence of GDM in mainland China was 14.8% in recent years, and relevant genetic knowledge for Chinese individuals is quite limited [27, 34–37]. To further identify the risk factors and mechanism of GDM in Chinese individuals at the genetic level, we conducted a GWAS based on a case-cohort design in Anqing, China.

## Methods

### Study subjects and phenotype diagnosis

The cohort enrolled 2232 pregnant women in the first/second trimester between February 2018 and December 2020 from Anqing Municipal Hospital in Anhui Province, China. Participants were followed up around their expected date of delivery. The demographical information, physical measurements, behavioural information before and during pregnancy, and medical histories of subjects were collected from structured questionnaires at baseline, structured questionnaires or phone interviews at follow-up, or medical records. The cohort profile is shown in (Additional file 1: Fig. S1). All subjects provided written informed consent when enrolled. Ethical approval for this study was obtained from the Medical Ethics Committee of Fudan University, School of Public Health (IRB00002408 & FWA00002399, Approval number: IRB#2017-09-0636).

A total of 203 GDM cases were identified using medical records, and these patients were diagnosed with GDM in the present pregnancy according to the *Chinese guidelines for diagnosis and treatment of gestational diabetes mellitus (2014)* by physicians. Pregnant women without diabetes before pregnancy met one or more of the following criteria: (a) blood glucose after 75 g OGTT  ≥ 10.0 mmol/L in the first hour or ≥ 8.5 mmol/L but < 11.1 mmol/L in second hour and (b) 5.1 mmol/L ≤ fasting blood glucose < 7.0 mmol/L after the 23rd gestational week (only for pregnant women who have no access to an OGTT) [28].

**Fig. 1:**
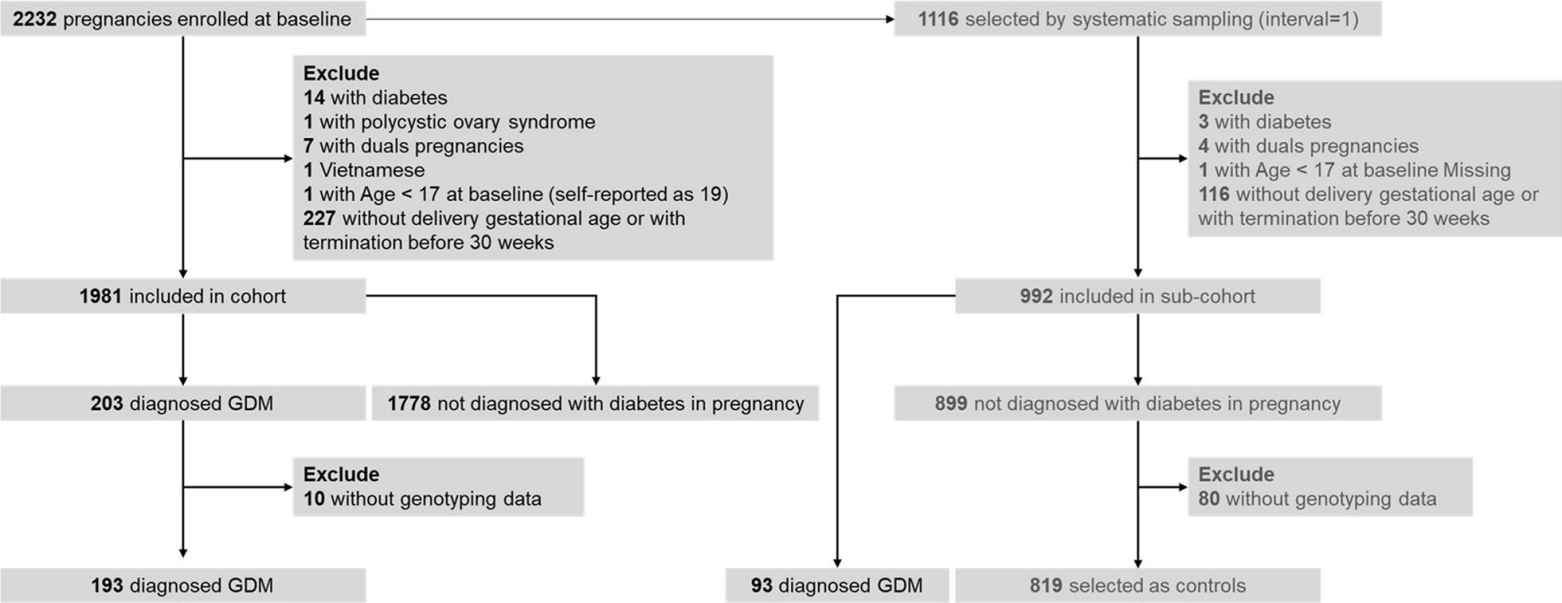
Flow diagram of this case-cohort study

A subcohort was sampled from the cohort by systematic sampling (interval = 1). Finally, 193 GDM patients (93 in the subcohort) and 819 subjects without diabetes diagnosis (controls) were selected for further analyses per the following criteria: singleton pregnancy, in the first/second trimester, aged 18 years or more, Chinese nationality, without a history of antibiotic use in the last four weeks at baseline, without systemic or organ diseases (such as diabetes, cardiovascular diseases, polycystic ovary syndrome) before pregnancy, with a gestational age of termination ≥ 30 weeks, and with genotyping data (Fig. 1).

### Biosample and genotyping

A 1.5-mL peripheral blood sample was collected from each subject at baseline for DNA extraction. All samples with a call rate > 95% were genotyped using an ASA-CHIA (Infinium Asian Screening Array-China Health Industry Alliance) Bead chip (Illumina, Inc., San Diego, California, USA) according to the Illumina Infinium HTS chip standard operating procedures (SOP) [38]. All genetic data sets used in this study were based on the human genome Hg19 assembly, and the rsIDs were mapped to dbSNP build 146 if necessary.

### Preprocessing of genotyped data

Single nucleotide polymorphisms (SNPs), insertions-deletions (InDels), and copy number variants (CNVs) were obtained using GenomeStudio version 2 according to Illumina GenomeStudio genotyping QC SOP v.1.6 [39].

#### Quality control (QC) and imputation for SNPs and InDels

For SNPs and InDels, QC was conducted per the following steps: merging the overlapped loci based on the Illumina GenTrain score (those with lower scores were excluded) and removing the loci on the Y chromosome using SAS 9.4. Subsequent QC steps were done following the guideline provided by Weale et al. [53] using PLINK [40] version 1.9 and R version 4.1.1, in which variants and individuals were excluded based on the following criteria: (1) individual and variant missingness > 0.02, (2) variants with minor allele frequency (MAF) < 0.05, (3) variants deviating from Hardy–Weinberg equilibrium (HWE) with P value < 1e− 10 in cases and < 1e− 6 in controls, (4) individuals deviating ± 3 standard deviation (SD) from the samples' heterozygosity rate mean, (5) individuals with a lower call rate from sample pairs with relatedness pi-hat value > 0.2, or (6) variants sharing the same coordinate and allele code. Genotype imputation was performed with the 1000 Genomes Project phase 3 East Asian population (1000G3 EAS) [41] using IMPUTE2 [42] for autosomal chromosomes and chromosome X. Only variants with info metric > 0.9, MAF < 0.01, missingness > 0.02, and unique coordinate and allele codes were kept.

#### Quality control and merging for CNVs

QC for CNV was performed by PennCNV [43] version 1.0.5 following the protocols provided by Lin et al. [44]. Signal intensity files obtained from GenomeStudio version 2 were split into one sample per file by column.pl script. The population frequency of the B allele file was compiled by compile_pfb.pl script. CNVs were called by the detect_cnv.pl script with a GCmodel adjustment to reduce false positive calls, and the cal_gc_snp.pl script was used to generate the customized GC model file. QC for called CNVs was conducted only for CNV calls with SNP numbers ≥ 10 by running filter_cnv.pl. Called CNVs in HLA and genomic regions near the centromeres or the telomeres within one million bases (Mb) were removed. CNVs with confidence scores < 10 and lengths > 5 Mb were further filtered by the HandyCNV R package [45]. Called CNVs from the same genomic locus can have various start and end points across individuals; thus, CNVs were merged into common CNV regions (CNVRs) by CNVRuler [46] version 1.2 trimming with CNV regional density < 0.1 and MAF < 0.05 to simplify the analysis.

### Annotating and prioritizing variants and genes from SNP/InDel-GWAS results in FUMA

Most hits from GWAS are in noncoding or intergenic regions and typically cannot be directly translated into causal variants; therefore, prioritizing the most likely causal variants and genes based on functional and biological knowledge is necessary. The FUMA [47] version 1.4.1 SNP2GENE process was used to analyse computed LD structure, to annotate functions to variants, to prioritize candidate genes based on functional gene mapping, and to perform gene-level analyses using GWAS summary statistics of variants with P values < 0.01. The total sample size was 985. Parameters were set as recommended by FUMA if not explained (Additional file 1: Note S1).

#### Characterizing genomic loci based on GWAS

Independent significant SNPs/InDels (ind. sig. markers) were defined as markers with P values ≤ 1e− 5 (suggestive significance level) and those that were independent of each other at r^2^ < 0.6, among which the lead markers were identified at r^2^ < 0.1. All known markers in the 1000G3 EAS reference panel or with P values < 0.05 in input GWAS statistics having r^2^ ≥ 0.6 with one of the ind. sig. markers (candidate markers) were included for further annotation. The LD blocks of ind. sig. markers located to each other within 250 kilobases (kb) were merged into one genomic locus. The data from the 1000G3 EAS were used to gauge the LD structure. Markers and genes in the MHC region were excluded for the complicated LD structure.

#### Functional gene mapping

Three strategies for gene mapping were applied to GWAS summary statistics with the following settings:

1. Positional mapping identifies the genes associated with candidate markers. The mapping was based on ANNOVAR annotations within a 10-kb physical distance between candidate markers and genes from Ensembl genes build 92.
2. Expression quantitative trait loci (eQTL) mapping maps markers to genes that likely affect the expression of those genes up to 1 Mb (cis-eQTL). The mapping was performed based on combined evidence from multiple eQTL data sources provided by FUMA (Additional file 1: Note S1). Only significant marker-gene pairs with a FDR P value < 0.05 were used.
3. Chromatin interactions (CI) regulate gene expression by bringing distal regulatory elements, such as superenhancers, to promoters in close spatial proximity. CI mapping was performed to map markers to genes if there was a three-dimensional DNA–DNA interaction between the marker region and another gene region without a distance boundary. For mapping databases, please see Additional file 1: Note S1. Interactions were filtered by a FDR P value < 1e− 6. Genes that were 250 bp upstream and 500 bp downstream of the transcription start site and that overlapped with the significantly interacting regions were mapped to further prioritize candidate genes. Predicted enhancer and promoter regions from the Roadmap epigenomics project for 111 epigenomes were also annotated to interaction regions.

#### Gene-based analysis by MAGMA

Gene-based analysis was performed for the input summary statistics using MAGMA version 1.08 in FUMA to increase the power of detecting genotype–phenotype associations. The Bonferroni correction was used to correct multiple testing of gene-based P values.

### Annotating genes for CNVRs

The HandyCNV R package was used to identify the genes located in each CNVR, and the reference gene panel was Human Release 19 version. CNVRs with a frequency of less than 0.05 were excluded because of their extremely high RRs and small P values.

### Overrepresentation analysis

WebGestalt [48] and g:Profiler [49] were used to perform overrepresentation analysis of genes. Genes mapped by more than two mapping methods (positional mapping, eQTL mapping, CI mapping, or Bonferroni corrected P value < 0.05 in MAGMA gene analysis) and annotated genes of significantly associated CNVRs were combined in these analyses as candidate genes. For parameters for WebGestalt and g:Profiler, please see (Additional file 1: Note S2, S3). Additional file 1: Fig. S2 shows the whole analysis strategy after variant calling.

### Statistical analyses

For continuous variables, the normality of distribution was tested by the one-sample Shapiro‒Wilk test (when sample size ≤ 2000) or Kolmogorov‒Smirnov test (when the sample size was > 2000). Continuous variables with a normal distribution are presented as the mean (SD), and those with a nonnormal distribution are presented as the median (interquartile range [IQR]). Means of 2 continuous normally distributed variables were compared by independent samples Student’s t-test. The Wilcoxon test and Kruskal‒Wallis test were used to compare the means of 2 and 3 or more groups of variables with nonnormal distributions, respectively. The frequencies of categorical variables were compared using Fisher’s exact test. For all statistical tests, a P value < 0.05 was considered significant [50]. All analyses above were performed in SAS 9.4 (SAS Institute Inc., NC, USA). Associations for SNPs, InDels, and CNVRs with GDM were estimated using the regression-based method for the case-cohort study proposed by Chui et al. [51]. The risk ratios (RRs), their 95% confidence intervals (CIs), and P values were reported. SNPs/InDels with a P value < 1e− 5 were defined as suggestively significant, and those with a P value < 1.61e− 7 (the empirical genome-wide significance threshold for SNP-GWAS in the East Asian population [52]) were defined as genome-wide significant. The study-wide P value threshold was defined as a false discovery rate (FDR)-corrected threshold assuming the whole false positive rate at 0.05 for CNVRs. All association analyses were performed in R 4.1.1.

According to the law of causality, the relation between genotypes and phenotypes is affected by only a few potential confounders, of which the most important is population stratification (PS). It can be estimated by the distinctions of observing genotypes among populations [79]. For SNPs/InDels, PS was calculated using the linkage-disequilibrium-pruned (LD-pruned) (window size 1500 variant count, step size 150 variant count, and pairwise r^2^ < 0.2) variant set on autosomal chromosomes [53, 54]. The top 50 principal components (PCs) of the variance-standardized relationship matrix were extracted using PLINK 1.9. Then, the “twstats” program of Eigensoft [55] version 6.1.4 was adopted to select the statistically significant PC axes (P value < 0.01) [53] to be included as covariates for the association analyses. Three PCs for CNVRs were selected using CNVRuler.

## Results

### Characteristics of participants and the representativeness of the subcohort

The median age of the study cohort was 28.0 years with an IQR of 25.0 to 30.0 years, and the median gestational age at baseline was 16.4 (IQR: 15.9–17.1) weeks. Approximately 99.2% (1953/1968) of the subjects in the cohort were of Han nationality. Balance tests for the subcohort group and nonselected group verified the representativeness of the sampling, in which the difference was significant only for the weight before pregnancy (P = 0.006) (Additional file 1: Table S1). Based on the case-cohort design and inclusion criteria, this study included 193 GDM patients and 912 subcohort subjects with 93 overlapping GDM cases between the two groups. The demographic characteristics of the study subjects are shown in Table 1.

**Table 1:** Demographic Characteristics of GDM cases and the sub-cohort in this study

Variables	GDM cases (N = 193)	Sub-cohort (N = 912)
Age at baseline, years	29.0 (26.0–30.0)	28.0 (25.0–30.0)
Baseline weight, kg	58.8 (53.0–66.3)	56.0 (51.0–62.0)
Weight before pregnancy, kg	56.0 (51.0–63.0)	54.0 (49.0–60.0)
Height, cm	160.0 (158.0–165.0)	160.0 (158.0–164.0)
Ethnic groups		
Han	192 (100.0%)	897 (99.0%)
Minority	0 (0.0%)	9 (1.0%)
Education		
Primary	4 (2.1%)	16 (1.8%)
Junior Secondary	38 (19.8%)	195 (21.5%)
Senior Secondary	40 (20.8%)	178 (19.6%)
College/university	110 (57.3%)	518 (57.1%)
Marriage		
Married	190 (98.4%)	889 (97.5%)
Unmarried or divorced	3 (1.6%)	23 (2.5%)
Family annual income, RMB		
Less than 100,000	65 (43.0%)	270 (37.3%)
100,000–200,000	69 (45.7%)	365 (50.5%)
200,000–300,000	11 (7.3%)	69 (9.5%)
More than 300,000	8 (4.0%)	19 (2.6%)
Active smoking		
Ceased after pregnancy	10 (5.2%)	26 (2.9%)
Current smoking	0 (0.0%)	2 (0.2%)
Never	182 (94.8%)	880 (96.9%)
Alcohol drinking		
Ceased after pregnancy	37 (19.5%)	151 (16.7%)
Current drinking	1 (0.5%)	1 (0.1%)
Never	152 (80.0%)	753 (83.2%)
Passive smoking every week		
0 day	123 (80.4%)	565 (77.3%)
1–3 days	14 (9.2%)	92 (12.6%)
4–5 days	6 (3.9%)	18 (2.5%)
6–7 days	10 (6.5%)	56 (7.7%)

Values are n (%), or median (interquartile range). There is missing data for some variables

*GDM* gestational diabetes, *RMB* renminbi

### SNPs/InDels associated with GDM and gene mapping

**Fig. 2:**
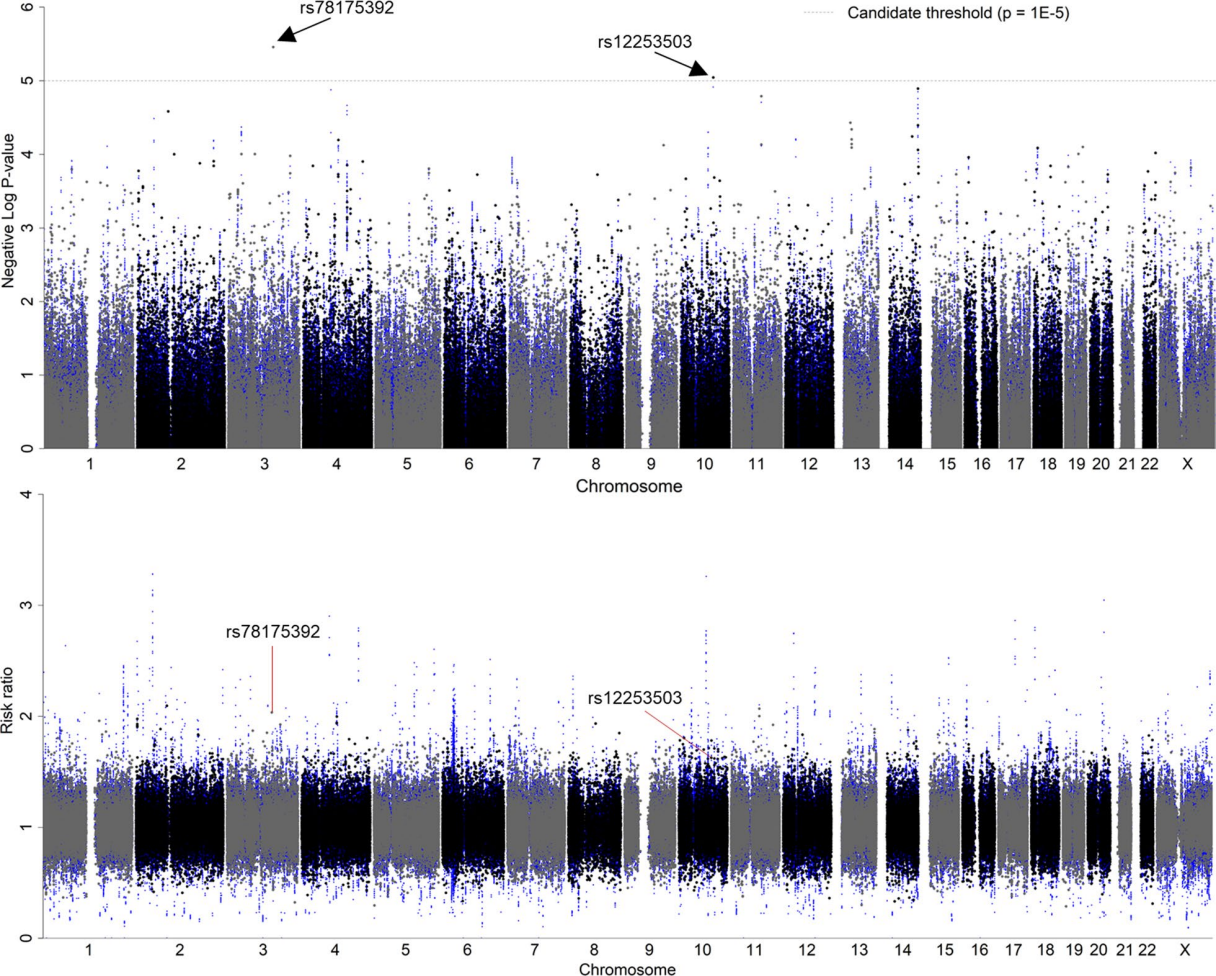
Manhattan plots of the P values and risk ratios reveal two independent significant markers. The blue points indicate imputed markers. The dotted line indicates the suggestive significance threshold (candidate threshold): P value < 1e− 5

A total of 1,544,258 SNPs and 123,886 InDels (referred to as “input markers”) from 985 subjects (190 cases and 795 controls) were included in the GWAS based on the imputation of 318,716 SNPs and 470 InDels. Three PCs were selected as covariates based on the “twstats” method (Additional file 1: Table S2). The inflation factor for the distribution of *P* values (Additional file 1: Fig. S3) was λ_GC_ = 1.01. There was no genome-wide significant marker, but two reached the suggestive significance level: SNP rs78175392:T:C (MAF: 0.14 in GDM group, 0.07 in non-DM group) in the intronic region of SLC12A8 gene on the chromosome (Chr) 3 (RR: 2.0 [1.5–2.7], *P* value: 3.5e− 06) and SNP rs12253503:A:G (MAF: 0.58 in GDM group, 0.45 in non-DM group) in the intergenic region of RP11-186O14.7 gene on Chr10 (RR: 1.6 [1.3–2.0], P value: 9.0e− 06) (Fig. 2, Additional file 1: Table S3). These two were defined as independent significant markers.

**Fig. 3:**
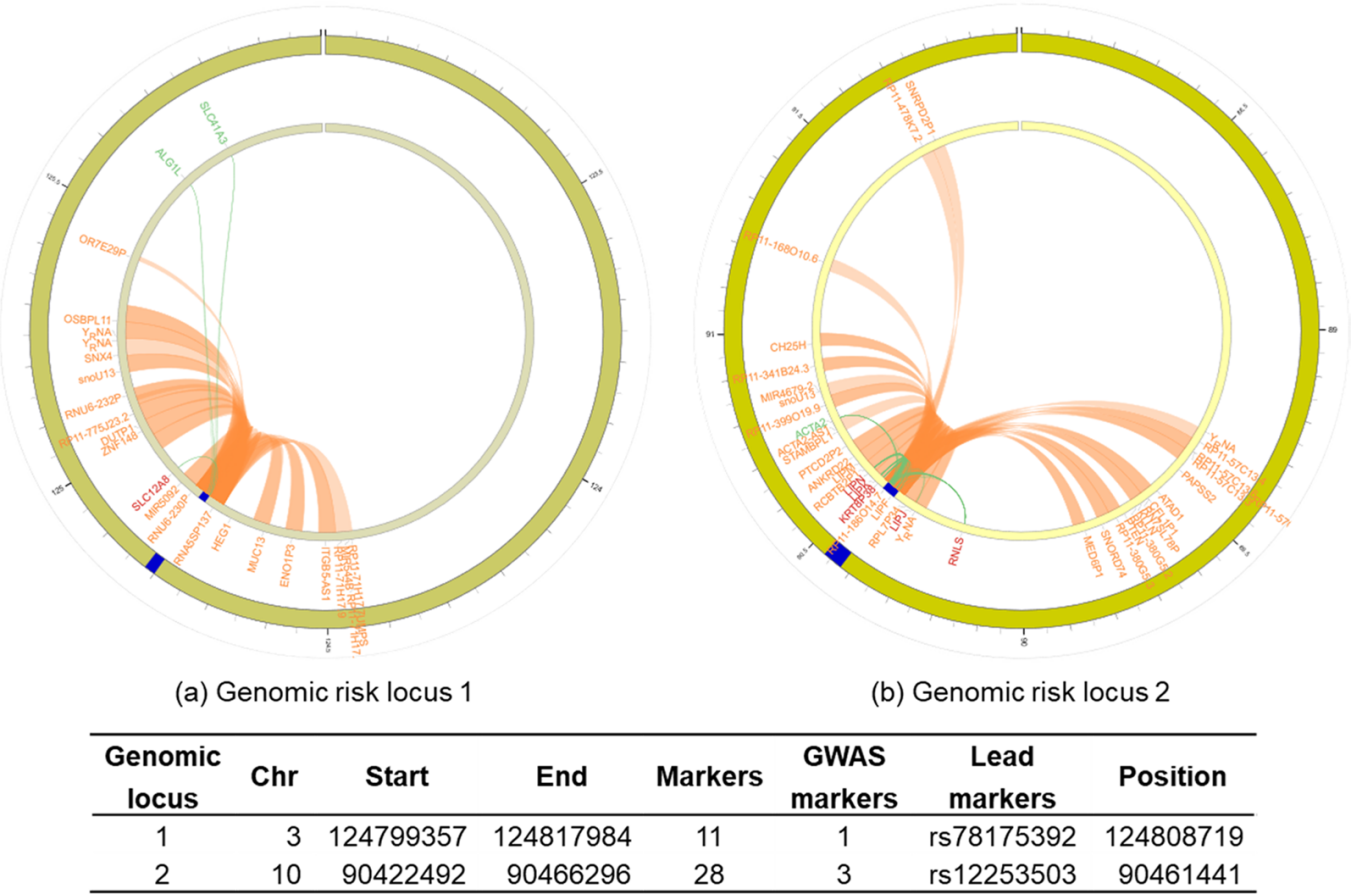
Circos plots of chromatin interactions and eQTLs mapping for SNPs/InDels. Genomic risk loci are highlighted in blue. Gene mapped only by chromatin interactions are colored orange, only by eQTLs are colored green, and mapped by both are colored red. Links colored orange are chromatin interactions and green are eQTLs. Chr = Chromosome; “Markers”, “GWAS markers”, and “Lead markers” mean the number of unique candidate markers, input GWAS-tagged candidate markers, and independent lead markers in the genomic locus, respectively

A total of 39 candidate markers (markers in LD with at least one independent significant marker at r^2^ ≥ 0.6) in two genomic risk loci (Fig. 3) were mapped to 64 genes by three mapping methods, including three by positional mapping (protein coding genes: SLC12A8 and LIPF; pseudogene: RP11-186O14.7), nine by eQTL mapping, and 61 genes by CI mapping (Additional file 1: Table S4). The SLC12A8 gene on Chr 3 and the RNLS, LIPJ, LIPK, KRT8P38, and LIPN genes on Chr 10 were mapped by eQTL mapping and CI mapping, and the LIPF and RP11-186O14.7 genes were also mapped by CI mapping (Fig. 3).

All input markers were mapped to 1007 protein coding genes in MAGMA gene-based analysis, and twelve reached the Bonferroni corrected significance threshold (Additional file 1: Table S5).

### Association analyses between CNVs and GDM and gene mapping

**Fig. 4:**
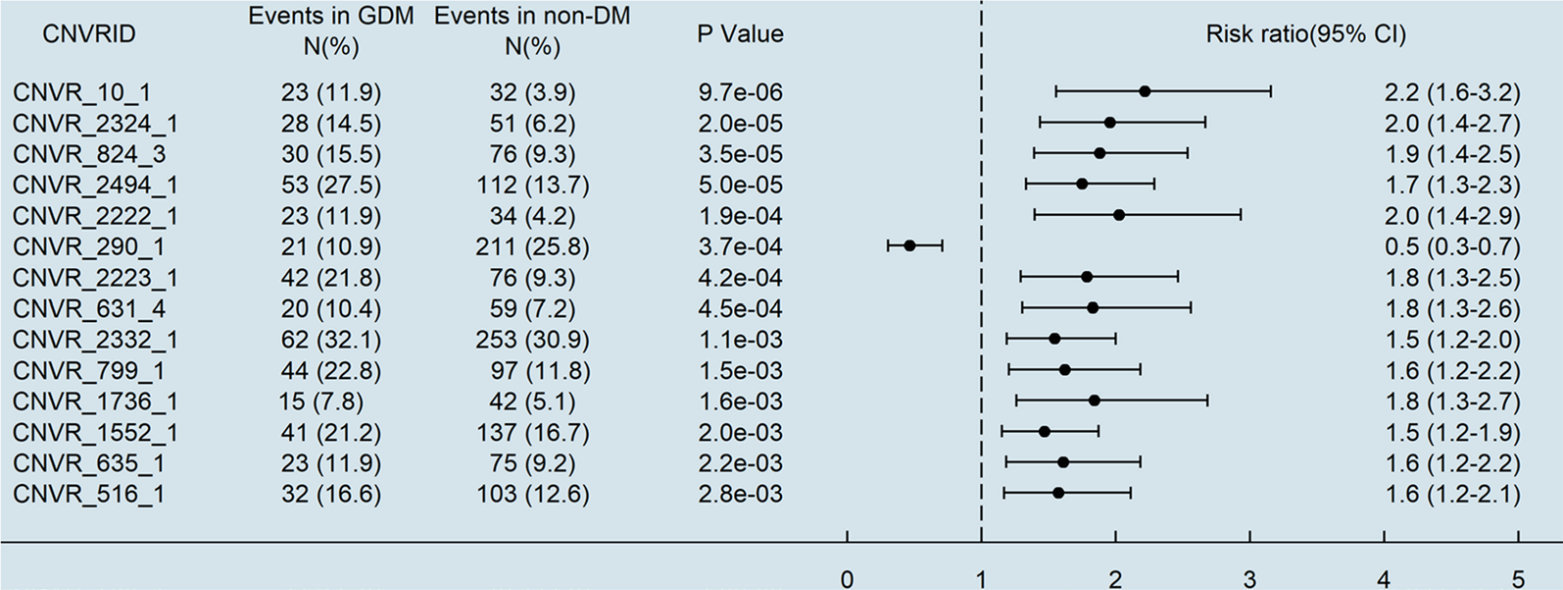
Association of CNVRs and GDM with false discovery rate corrected P value < 0.05. *CNVR*  copy number variant region, *DM*  diabetes mellitus, *GDM*  gestational diabetes mellitus

**Fig. 5:**
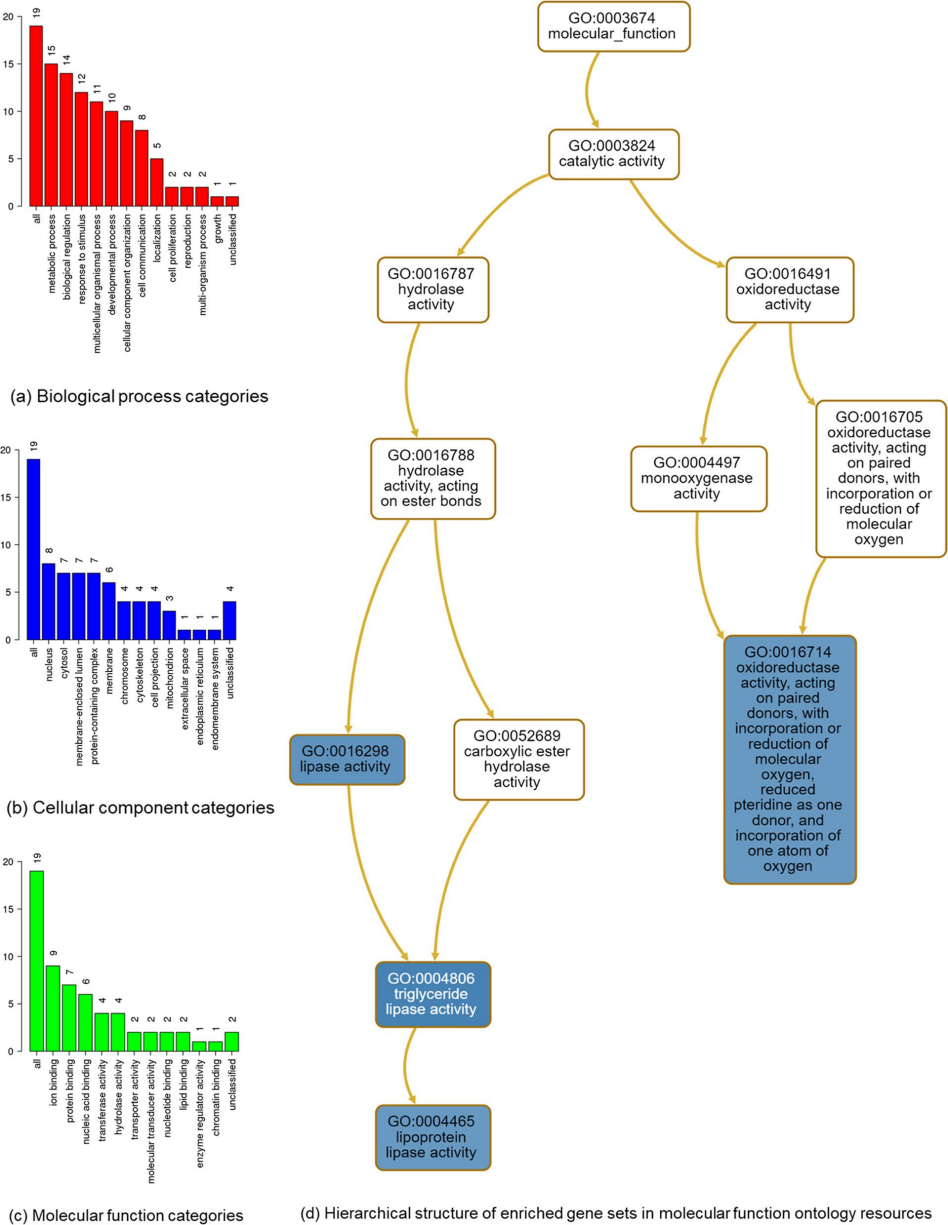
Categories of input genes and enriched gene sets of molecular function ontologies in WebGestalt analysis. **a**–**c** Categories of input genes **d** Gene sets highlighted in blue were significantly enriched in GO database

A total of 51,688 CNVs were detected and filtered by PennCNV. These CNVs were merged into 220 CNVRs that were included in the association analyses with three PC covariates. Fourteen CNVRs were defined as significantly associated with GDM through FDR correction (Fig. 4) and mapped to 12 genes (Table 2, Additional file 1: Table S6) (Fig. 5).

**Table 2:** Candidate genes for gene over-representation analyses

Gene	Gene name	Chr	Type	Primary molecular function
APC	APC, WNT signaling pathway regulator	5	Protein coding	Tumor suppressor
BRCA1	BRCA1, DNA repair associated	17	Protein coding	Facilitating cellular responses to DNA damage
CLOCK	Clock circadian regulator	4	Protein coding	Transcriptional activator as a core component of the circadian clock
GRIN3B	Glutamate ionotropic receptor nmda type subunit 3b	19	Protein coding	N-methyl-D-aspartate receptor subtype of glutamate-gated ion channels with reduced single-channel conductance, low calcium permeability and low voltage-dependent sensitivity to magnesium
GSK3B	Glycogen synthase kinase 3 beta	3	Protein coding	A negative regulator in the hormonal control of glucose homeostasis, Wnt signaling and regulation of transcription factors and microtubules
KRT8P38^M^	Keratin 8 pseudogene 38	10	Pseudogene	NA
LIPF^M^	Lipase F, gastric type	10	Protein coding	Catalyzing the hydrolysis of triacylglycerols to free fatty acids, diacylglycerol, monoacylglycerol, and glycerol
LIPJ^M^	Lipase family member J	10	Protein coding	Enabling hydrolase activity, acting on ester bonds
LIPK^M^	Lipase family member K	10	Protein coding	Playing a highly specific role in the last step of keratinocyte differentiation; may have an essential function in lipid metabolism of the most differentiated epidermal layer
LIPN^M^	Lipase family member N	10	Protein coding	Same as the LIPK
NR3C1	Nuclear receptor subfamily 3 group C member 1	5	Protein coding	Receptor for glucocorticoids
PRDM16	PR/SET domain 16	1	Protein coding	Binds DNA and functions as a transcriptional regulator
RNLS^M^	Renalase, FAD dependent amine oxidase	10	Protein coding	Catalyzing the oxidation of the less abundant 1,2-dihydro-beta-NAD(P) and 1,6-dihydro-beta-NAD(P) to beta-NAD(P)( +)
SALL3	Spalt like transcription factor 3	18	Protein coding	Probable transcription factor
SLC12A8^S^	Solute carrier family 12 member 8	3	Protein coding	Ation/chloride cotransporter that may play a role in the control of keratinocyte proliferation
SYCP2	Synaptonemal complex protein 2	20	Protein coding	Major component of the axial/lateral elements of synaptonemal complexes during meiotic prophase
TMEM259	Transmembrane protein 259	19	Protein coding	May have a role in the endoplasmic-reticulum-associated protein degradation (ERDA) pathway required for clearance of misfolded proteins in the ER; promoting survival of motor neurons
TPH1	Tryptophan hydroxylase 1	11	Protein coding	Oxidizing L-tryptophan to 5-hydroxy-l-tryptophan in the rate-determining step of serotonin biosynthesis
TPH2	Tryptophan hydroxylase 2	12	Protein coding	Enabling tryptophan 5-monooxygenase activity

Genes with superscript “S” were mapped by SNPs associated with GDM from GWAS, genes with superscript “M” were mapped by mapping strategies using GWAS summary statistics, and others were mapped by CNVRs directly associated with GDM. Molecular function of genes is from the GeneCards databases

*Chr* chromosome, *NA* not applicable

### Overrepresentation analyses (ORA) of mapped genes

**Fig. 6:**
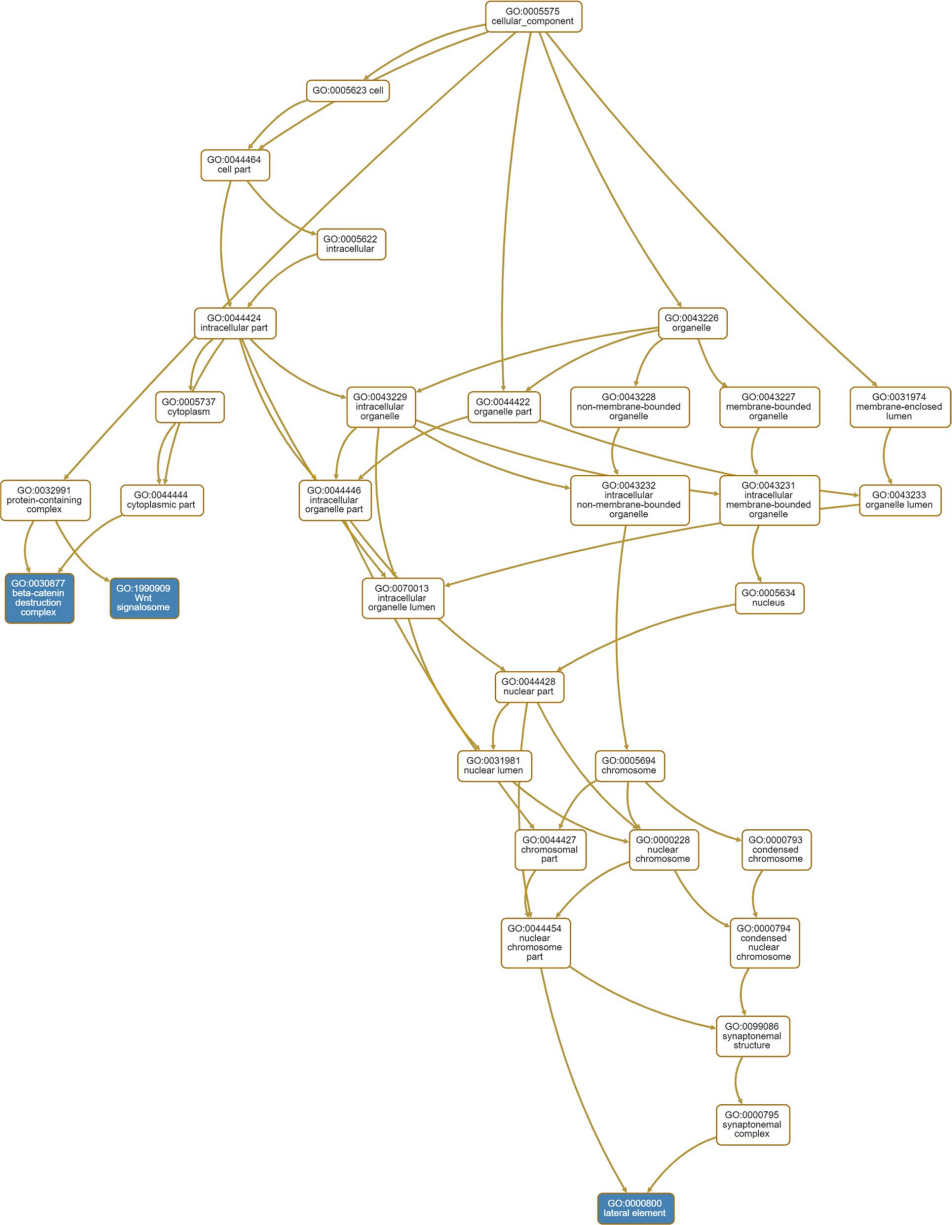
Hierarchical structure of enriched gene sets of cellular component ontologies in WebGestalt analysis. Gene sets highlighted in blue were significantly enriched in GO database

Seven genes mapped by at least two mapping methods in the SNP/InDel part (with RP11-186O14.7 gene removed because of a lack of information in all available resources) and 12 genes mapped in the CNV part were included in the ORA as candidate genes of GDM. The 19 genes included 18 protein-coding genes and 1 pseudogene, and their primary molecular functions are shown in Table 2. Details of the genes can be found in the NCBI and GeneCards databases (Additional file 1: Table S7). In WebGestalt analysis, the 19 candidate genes were mapped to 19 unique Entrezgene IDs and were classified into different biological process, cellular component, and molecular categories (Fig. 5a–c). For biological process categories, 15 genes were related to metabolic processes, and 14 were related to biological regulation. Most of these 19 genes were associated mainly with molecular binding processes, such as ion binding (N = 9), protein binding (N = 7), and nucleic acid binding (N = 6). The ORA of genes identified 7 gene sets in WebGestalt (Table 3) and 16 gene sets (Table 3, Additional file 1: Table S8) in g:Profiler. Gene sets enriched by WebGestalt were related to lipase activity (including the LIPF, LIPN, LIPK, and LIPJ genes; Table 3), oxidoreductase activity (including the TPH1 and TPH2 genes; Table 3) and molecular function (Fig. 5d) and were connected with 3 cellular components, namely, the beta-catenin destruction complex, Wnt signalosome, and lateral element (Fig. 6).  The descriptions and definitions of these 7 gene sets are shown in Table 3. The g:Profiler also identified these 7 gene sets, in addition to one related to tryptophan 5-monooxygenase activity and eight related to biological processes, including the serotonin biosynthetic process, indole-containing compound biosynthetic process, primary amino compound biosynthetic process, positive regulation of protein localization to centrosome, cornification, regulation of type B pancreatic cell development, circadian rhythm, and regulation of protein localization to centrosome (Additional file 1: Table S8).

**Table 3:** Gene sets enriched in the Gene Ontology resource by the WebGestalt and g:Profiler

Gene set	Description	Type	Gene	P_FDR_	P_adj_	Definition
GO:0,004,806	Triglyceride lipase activity	MF	LIPF LIPK LIPN	0.003	0.000	Catalysis of the hydrolysis of triglyceride to diglyceride
GO:0,016,298	Lipase activity	MF	LIPF LIPJ LIPK LIPN	0.007	0.048	Catalysis of the hydrolysis of a lipid or phospholipid
GO:0,004,465	Lipoprotein lipase activity	MF	LIPK LIPN	0.010	0.008	Catalysis of the hydrolysis of triglyceride in lipoprotein to diglyceride
GO:0,016,714	Oxidoreductase activity	MF	TPH1 TPH2	0.010	0.004	Catalysis of an oxidation–reduction reaction
GO:0,030,877	Beta-catenin destruction complex	CC	APC GSK3B	0.013	0.008	A cytoplasmic protein complex containing glycogen synthase kinase-3-beta
GO:1,990,909	Wnt signalosome	CC	APC GSK3B	0.013	0.008	A multiprotein protein complex containing membrane-localized Wnt receptors and cytosolic protein complexes
GO:0,000,800	Lateral element	CC	BRCA1 SYCP2	0.013	0.011	A proteinaceous core found between sister chromatids during meiotic prophase

*GO* gene ontology, *P*_*FDR*_ P value corrected by false discovery rate method in WebGestalt analysis. P_adj_ = P value corrected by g:SCS method which corresponds to an experiment-wide threshold of α = 0.05 in g:Profiler analysis

*CC* cellular component, *MF* molecular function

## Discussion

In this case-cohort GWAS based on SNP array genotyping and imputing data, we aimed to explore the genetic background of GDM in a Chinese population and identified 2 SNPs associated with GDM at a suggestive significance level and 14 CNVRs associated with GDM. A total of 19 genes that may be candidate genetic markers for GDM were mapped through functional mapping, in which twelve (APC, BRCA1, CLOCK, GRIN3B, GSK3B, NR3C1, PRDM16, SALL3, SYCP2, TMEM259, TPH1, and TPH2) were directly mapped by variants genotyped in our study. These 19 candidate genes were enriched mainly in gene sets related to the triglyceride lipase (TGL) activity and lipoprotein lipase (LPL) activity, oxidoreductase activity, and the cellular components beta-catenin destruction complex, Wnt signalosome, and lateral element based on the GO resource.

TGL is a family of lipases that catalyse the first stage of lipolysis by hydrolysing triglyceride (also termed “triacylglycerol” in recent literature, TG) to diacylglycerol (DAG) and fatty acid (FA), while LPL is responsible for hydrolysing TG in blood lipoproteins to DAG and FA [56]. Convincing evidence indicates an association between lipolysis and IR. Kim et al. found that transgenic mice with tissue-specific overexpression of LPL have increased TG and IR in a specific tissue, and there is a causative relationship between the accumulation of intracellular TG and IR [57]. Inhibition of TGL in adipose tissue improves whole-body insulin sensitivity [56, 58–60]. DAG acts as a direct activator of many kinases that impair insulin receptor substrates and insulin signalling [61]. Girousse et al. revealed that a high lipolytic rate was associated with low insulin sensitivity in human and partial genetic and pharmacologic inhibition of hormone-sensitive lipase (an lipase intracellular lipolysis) resulted in the improvement of insulin sensitivity in mice [62], which also supported that lipolysis could affect the insulin sensitivity. Since insulin is the main factor inhibiting lipolysis, decreasing insulin sensitivity and increasing lipolysis in later pregnancy could result in a vicious cycle [58, 63]. Oxidoreductase is a class of enzymes catalysing oxidoreduction reactions (redox), which play an important role in human metabolism [64]. Excessive FA, DAG, and other lipid metabolites could cause an increasing level of free FA (FFA), redox imbalance, a decrease in oxidative capacity in adipose tissue and then excessive ectopic lipid deposits in the muscle, liver, and pancreas, which further aggravates IR and hyperglycaemia [61]. T2DM has been recently postulated to be a redox disease, while reliable evidence is absent [65]. The Wnt signalosome is formed to increase the local concentration and signalling activity of Wnt signals [66]. Beta-catenin is a functional protein that can regulate the expression of Wnt target genes and is the key nuclear effector of the Wnt signalling pathway [67, 68]. Wnt signalling and β-catenin are both necessary and sufficient for the proliferation of islet β-cells [69, 70]. Fair evidence indicates that Wnt/β-catenin signalling favours improved insulin/glucose and lipid homeostasis and that antagonism of this pathway by oxidative stress may contribute to IR and hyperlipidaemia [68]. Conventional dendritic cells with constitutively activated β-catenin induce islet expansion by increasing b-cell proliferation in a mouse model of diet-induced obesity [71]. Beta-catenin signalling could also regulate preadipocyte differentiation, and given that obesity is one of the main risk factors for diabetes, it is conceivable that changes in components of the Wnt signalling pathway could contribute to an increased risk of diabetes by impacting adipogenesis [72]. Taken together, these findings suggest that the onset of GDM may be the result of asynchrony of metabolic changes in pregnancy, in which the insulin signalling pathway and glycometabolism are affected based on the genetic background related to lipometabolism and redox.

To our knowledge, only two GWASs of GDM have been reported. Kwak et al. conducted a two-stage study in Korean women, including 468 GDM cases and 1242 nondiabetic controls using 2.19 million genotyped or imputed markers in the first stage and 931 GDM cases and 783 nondiabetic controls with 11 genotyping loci from the first stage in further study [26]. Nine independent significant SNPs (P value < 2e− 5 and LD r^2^ < 0.5) were identified in the GWAS, among which rs7754840 in the intron regions of the CDKAL1 gene and rs10830962 located upstream of the MTNR1B gene showed the strongest association with GDM. The controls were selected from women without a T2DM history  ≥ 50 years in the first stage and  ≥ 50 years in the second stage, which may introduce misclassification bias because of an unknown GDM history. The second GWAS included 103 GDM cases and 115 controls from a population of Chinese Han women and identified 23 SNPs mapping to four genes (CTIF, CDH18, PTGIS, and SYNPR) [27]. No overlapping SNP markers or mapped genes exist among the two and the present study. There are many differences between the three GWASs, including different populations, GDM diagnosis strategies, sample sizes, genotyping chips, imputing methods, and analysis strategies, details are shown in Additional file 1: Table S8; therefore, it is difficult to compare the results from these GWASs, so more GWASs of GDM are needed. Most genetic studies for GDM in humans are candidate gene studies based on other types of diabetes, particularly T2DM. Some genes directly associated with GDM in our study, such as CLOCK [73], NR3C1 [74], and PRDM16 [75], mainly in the European population, have also been reported to be associated with T2DM. However, gene variants associated with a specific phenotype could vary among ethnicities, districts, genetic markers selected, and even sample sizes. It is difficult to define the causality of a genetic variant before more work is done to verify its association with a phenotype in a specific population, so we focused more on explanations of the combined biological functions of associated genes.

GDM has been a public health problem with both short-term and long-term metabolic influences on mothers and children. Converging lines of evidence have shaped GDM as a complex trait caused by genetic and environmental risk factors. However, the pathophysiology of GDM remains unclear and controversial, and its genetics have not been fully explored. Our study suggests that GDM may be the result of alterations in energy metabolism under a specific genetic background, including gene variants related to lipolysis and redox in addition to the Wnt signalling pathway, which affect glucose metabolism mediated by the insulin signalling pathway. This finding adds evidence for the hypothesis that GDM may be a herald of T2DM expressed under the specific metabolic conditions of pregnancy or an excessive manifestation of metabolic alterations occurring in pregnancy [76] and explains why obesity is a risk factor for GDM at the gene level. With changes in maternal homeostasis to contain the growing foetus, pregnancy represents a unique metabolic state and imposes many metabolic challenges on the mother, making it easier to develop metabolic diseases such as diabetes. This study could provide more insights into the prevention and treatment of GDM by highlighting the importance of lipid storage and metabolism in pregnancy, such as using lipid metabolites as an early screening biomarker for GDM or intervention target to manage GDM. Additionally, instrumental variables could be built based on the genetic variants associated with GDM to estimate the associations between GDM and related outcomes, which could help control the confounding bias in epidemiologic studies [77, 78].

The strengths of the present study are as follows. (1) This study was based on a case-cohort study design, which is cost-effective for GWAS and could produce RR estimates without the rare-disease assumption [79]. (2) Genome-wide SNP array genotyping data imputed with 1000G3 are also cost-effective in exploring the genetic risk factors for a specific phenotype from the whole genome level. (3) Compared to other GWASs of GDM, we analysed the association between genetic variants and GDM at the SNP, InDel, and CNV levels to fully clarify our research questions. (4) To obtain reliable results, we conducted QC of genotyping data according to published guidelines and performed functional analyses using as many parallel methods as we could. However, our study has some inevitable limitations. (1) The majority of subjects enrolled were Han in Anqing, China, and GDM diagnosis is usually different among districts. Thus, the generalizability of our results may be limited because of the population’s genetic specificity and the nonuniform phenotype definition. (2) GWAS could detect only associations and not causality, and the biological knowledge of hits and loci in LD with them for functional analyses was obtained mainly from the Caucasian population. (3) Furthermore, gene expression is complicated and influenced by the environment. Thus, the results from our study cannot determine the causal genetic variants of GDM. (4) While SNP-based GWAS has standardized processes, there are few conventions for CNV analysis. We performed ORA rather than gene set enrichment analysis for the unavailable ranked scores for genes annotated from CNVRs. (5) No genome-wide significant variant was identified in our study, and we might have missed genetic variants specific to GDM but with a small effect with a small sample size for a GWAS. In summary, our results need verification from more GWASs and other mechanistic studies (such as epigenetic, proteomic, metabolomic, pathway, molecular, and animal model studies) of GDM in the Chinese population.

## Conclusions

This GWAS of GDM in a sample of Chinese women showed evidence that GDM is a metabolic disease and that lipid metabolism, particularly lipolysis, may play an important role in the onset and development of GDM. The results provide a new direction for the prevention, early diagnosis, and treatment of GDM, focusing more on metabolites other than blood glucose. However, further studies, such as GWASs in other populations or studies using other omics, animal models, or GDM-associated genes from this study, should be conducted to verify and detail the actual mechanism of these hypotheses.

## Supplementary Information

**Additional file 1. Note S1.** Parameters for FUMAGWAS analyses. **Note S2.** Parameters for WebGestalt analyses. Note S3. Parameters for g:profiler analyses. **Table S1.** Characteristics of selected or not sub-cohort groups in cohort. **Table S2.** PCs with P value < 0.01 for SNPs/InDels association analyses. **Table S3.** Markers in linkage disequilibrium with any independent significant markers (r2 ≥ 0.6). **Table S4.** Genes mapped by positional mapping, eQTL mapping, and chromatin interaction mapping. **Table S5.** Protein coding genes from MAGMA gene-based analyses with PBon < 0.05. **Table S6.** CNVRs associated with GDM FDR with p value < 0.05. **Table S7.** Candidate genes for gene over-representation analyses. **Table S8.** Gene sets enriched in the Gene Ontology resource by g: Profiler. **Table S9.** Genome-wide association studies of GDM reported in literatures and the present study. **Figure S1.** Profile of the study cohort. **Figure S2.** Strategies of the analyses. **Figure S3.** The quantile–quantile plot of P values of SNPs/InDels GWAS (λ=1.01).

## Data Availability

The datasets analysed during the current study are not publicly available due to the *Regulation of the People's Republic of China on the Administration of Human Genetic Resources*, but the summary statistics of datasets are available from the corresponding author on reasonable request.

## Abbreviations

CIs: Confidence intervals
CNV: Copy number variant
CNVR: Copy number variants region
CVD: Cardiovascular diseases
DAG: Diacylglycerol
eQTL: Expression quantitative trait loci
FDR: False discovery rate
FFA: Free fatty acid
GDM: Gestational diabetes mellitus
GWAS: Genome-wide association study
HWE: Hardy–Weinberg equilibrium
InDel: Insertions-deletion
IR: Insulin resistance
kb: Kilobases
LD: Linkage-disequilibrium
MAF: Minor allele frequency
MS: Metabolic syndrome
PC: Principal component
PS: Population stratification
QC: Quality control
RR: Risk ratio
SNP: Single nucleotide polymorphism
SOP: Standard operating procedure
T2DM: Type 2 diabetes mellitus
TG: Triglyceride
