# Supplementary material for: Lipolysis and gestational diabetes mellitus onset: a case-cohort genome-wide association study in Chinese
Source: J Transl Med. 2023 Jan 25;21:47. doi: 10.1186/s12967-023-03902-4 (PMC9875546; doi:10.1186/s12967-023-03902-4)
Supplement: Supplementary file 1 — Additional file 1. Note S1. Parameters for FUMAGWAS analyses. Note S2. Parameters for WebGestalt analyses. Note S3. Parameters for g:profiler analyses. Table S1. Characteristics of selected or not sub-cohort groups in cohort. Table S2. PCs with P value < 0.01 for SNPs/InDels association analyses. Table S3. Markers in linkage disequilibrium with any independent significant markers (r2 ≥ 0.6). Table S4. Genes mapped by positional mapping, eQTL mapping, and chromatin interaction mapping. Table S5. Protein coding genes from MAGMA gene-based analyses with PBon < 0.05. Table S6. CNVRs associated with GDM FDR with p value < 0.05. Table S7. Candidate genes for gene over-representation analyses. Table S8. Gene sets enriched in the Gene Ontology resource by g: Profiler. Table S9. Genome-wide association studies of GDM reported in literatures and the present study. Figure S1. Profile of the study cohort. Figure S2. Strategies of the analyses. Figure S3. The quantile–quantile plot of P values of SNPs/InDels GWAS (λ=1.01). [file 12967_2023_3902_MOESM1_ESM.docx]

# Lists of Additional file

[Additional file Notes 2](#_Toc120111538)

[Note S1. Parameters for FUMAGWAS analyses 2](#_Toc120111539)

[Note S2. Parameters for WebGestalt analyses 7](#_Toc120111540)

[Note S3. Parameters for g:profiler analyses 8](#_Toc120111541)

[Additional file Tables 9](#_Toc120111542)

[Table S1. Characteristics of selected or not sub-cohort groups in cohort 9](#_Toc120111543)

[Table S2. PCs with P value < 0.01 for SNPs/InDels association analyses 11](#_Toc120111544)

[Table S3. Markers in linkage disequilibrium with any independent significant markers (r^2^ ≥ 0.6) 12](#_Toc120111545)

[Table S4. Genes mapped by positional mapping, eQTL mapping, and chromatin interaction mapping 14](#_Toc120111546)

[Table S5. Protein coding genes from MAGMA gene-based analyses with P_Bon_ < 0.05 17](#_Toc120111547)

[Table S6. CNVRs associated with GDM FDR with p value < 0.05 18](#_Toc120111548)

[Table S7. Candidate genes for gene over-representation analyses 19](#_Toc120111549)

[Table S8. Gene sets enriched in the Gene Ontology resource by g: Profiler 20](#_Toc120111550)

[Table S9. Genome-wide association studies of GDM reported in literatures and the present study 23](#_Toc120111551)

[Additional file Figures 25](#_Toc120111552)

[Figure S1. Profile of the study cohort 25](#_Toc120111553)

[Figure S2. Strategies of the analyses 25](#_Toc120111554)

[Figure S3. The quantile–quantile plot of P values of SNPs/InDels GWAS (λ=1.01) 26](#_Toc120111555)

# Additional file Notes

Note S1. Parameters for FUMAGWAS analyses

**[jobinfo]**

created_at = 2022-09-23 04:55:28

title = GDMpaperpost

**[version]**

FUMA = v1.4.1

MAGMA = v1.08

GWAScatalog = e104_r2021-09-15

ANNOVAR = 2017-07-17

**[inputfiles]**

gwasfile = FUMA_GDM_rs.txt

chrcol = NA

poscol = NA

rsIDcol = NA

pcol = NA

eacol = NA

neacol = NA

orcol = or

becol = beta

secol = se

leadSNPsfile = NA

addleadSNPs = 1

regionsfile = NA

**[params]**

N = 985

Ncol = NA

exMHC = 1

MHCopt = annot

extMHC = NA

ensembl = v92

genetype = all

leadP = 1e-5

gwasP = 0.05

r2 = 0.6

r2_2 = 0.1

refpanel = 1KG/Phase3

pop = EAS

MAF = 0

refSNPs = 1

mergeDist = 250

**[magma]**

magma = 1

magma_window = 0

magma_exp = GTEx/v8/gtex_v8_ts_avg_log2TPM:GTEx/v8/gtex_v8_ts_general_avg_log2TPM:BrainSpan/bs_age_avg_log2RPKM:BrainSpan/bs_dev_avg_log2RPKM

**[posMap]**

posMap = 1

posMapWindowSize = 10

posMapAnnot = NA

posMapCADDth = 0

posMapRDBth = NA

posMapChr15 = NA

posMapChr15Max = NA

posMapChr15Meth = NA

posMapAnnoDs = NA

posMapAnnoMeth = NA

**[eqtlMap]**

eqtlMap = 1

eqtlMaptss = EyeGEx/EyeGEx.txt.gz:eQTLcatalogue/Alasoo_2018_ge_macrophage_IFNg_Salmonella.txt.gz:eQTLcatalogue/Alasoo_2018_ge_macrophage_IFNg.txt.gz:eQTLcatalogue/Alasoo_2018_ge_macrophage_naive.txt.gz:eQTLcatalogue/Alasoo_2018_ge_macrophage_Salmonella.txt.gz:eQTLcatalogue/BLUEPRINT_ge_monocyte.txt.gz:eQTLcatalogue/BLUEPRINT_ge_neutrophil.txt.gz:eQTLcatalogue/BLUEPRINT_ge_T-cell.txt.gz:eQTLcatalogue/BrainSeq_ge_brain.txt.gz:eQTLcatalogue/CEDAR_B-cell_CD19.txt.gz:eQTLcatalogue/CEDAR_ileum.txt.gz:eQTLcatalogue/CEDAR_monocyte_CD14.txt.gz:eQTLcatalogue/CEDAR_neutrophil_CD15.txt.gz:eQTLcatalogue/CEDAR_platelet.txt.gz:eQTLcatalogue/CEDAR_rectum.txt.gz:eQTLcatalogue/CEDAR_T-cell_CD4.txt.gz:eQTLcatalogue/CEDAR_T-cell_CD8.txt.gz:eQTLcatalogue/CEDAR_transverse_colon.txt.gz:eQTLcatalogue/Fairfax_2012_B-cell_CD19.txt.gz:eQTLcatalogue/Fairfax_2014_IFN24.txt.gz:eQTLcatalogue/Fairfax_2014_LPS24.txt.gz:eQTLcatalogue/Fairfax_2014_LPS2.txt.gz:eQTLcatalogue/Fairfax_2014_naive.txt.gz:eQTLcatalogue/GENCORD_ge_fibroblast.txt.gz:eQTLcatalogue/GENCORD_ge_LCL.txt.gz:eQTLcatalogue/GENCORD_ge_T-cell.txt.gz:eQTLcatalogue/GEUVADIS_ge_LCL.txt.gz:eQTLcatalogue/HipSci_ge_iPSC.txt.gz:eQTLcatalogue/Kasela_2017_T-cell_CD4.txt.gz:eQTLcatalogue/Kasela_2017_T-cell_CD8.txt.gz:eQTLcatalogue/Lepik_2017_ge_blood.txt.gz:eQTLcatalogue/Naranbhai_2015_neutrophil_CD16.txt.gz:eQTLcatalogue/Nedelec_2016_ge_macrophage_Listeria.txt.gz:eQTLcatalogue/Nedelec_2016_ge_macrophage_naive.txt.gz:eQTLcatalogue/Nedelec_2016_ge_macrophage_Salmonella.txt.gz:eQTLcatalogue/Quach_2016_ge_monocyte_IAV.txt.gz:eQTLcatalogue/Quach_2016_ge_monocyte_LPS.txt.gz:eQTLcatalogue/Quach_2016_ge_monocyte_naive.txt.gz:eQTLcatalogue/Quach_2016_ge_monocyte_Pam3CSK4.txt.gz:eQTLcatalogue/Quach_2016_ge_monocyte_R848.txt.gz:eQTLcatalogue/Schwartzentruber_2018_ge_sensory_neuron.txt.gz:eQTLcatalogue/TwinsUK_ge_blood.txt.gz:eQTLcatalogue/TwinsUK_ge_fat.txt.gz:eQTLcatalogue/TwinsUK_ge_LCL.txt.gz:eQTLcatalogue/TwinsUK_ge_skin.txt.gz:eQTLcatalogue/van_de_Bunt_2015_ge_pancreatic_islet.txt.gz:PsychENCODE/PsychENCODE_eQTLs.txt.gz:scRNA_eQTLs/B_cell.txt.gz:scRNA_eQTLs/DC.txt.gz:scRNA_eQTLs/NK.txt.gz:scRNA_eQTLs/Monocyte.txt.gz:scRNA_eQTLs/Classical_Monocyte.txt.gz:scRNA_eQTLs/Non_classical_Monocyte.txt.gz:scRNA_eQTLs/T_CD4.txt.gz:scRNA_eQTLs/T_CD8.txt.gz:scRNA_eQTLs/PBMC.txt.gz:DICE/B_cell_naive.txt.gz:DICE/T_CD4_naive.txt.gz:DICE/T_CD4_naive_activated.txt.gz:DICE/T_CD8_naive.txt.gz:DICE/T_CD8_naive_activated.txt.gz:DICE/Monocyte_classical.txt.gz:DICE/Monocyte_non_classical.txt.gz:DICE/NK.txt.gz:DICE/T_CD4_TFH.txt.gz:DICE/T_CD4_TH1.txt.gz:DICE/T_CD4_TH17.txt.gz:DICE/T_CD4_TH1_17.txt.gz:DICE/T_CD4_TH2.txt.gz:DICE/T_CD4_memory_TREG.txt.gz:DICE/T_CD4_naive_TREG.txt.gz:eQTLGen/eQTLGen_cis_eQTLs.txt.gz:eQTLGen/eQTLGen_trans_eQTLs.txt.gz:BloodeQTL/BloodeQTL.txt.gz:BIOSQTL/BIOS_eQTL_geneLevel.txt.gz:MuTHER/MuTHER_Adipose.txt.gz:MuTHER/MuTHER_LCL.txt.gz:MuTHER/MuTHER_Skin.txt.gz:xQTLServer/xQTLServer_eQTLs.txt.gz:CMC/CMC_SVA_cis.txt.gz:CMC/CMC_SVA_trans.txt.gz:CMC/CMC_NoSVA_cis.txt.gz:CMC/CMC_NoSVA_trans.txt.gz:BRAINEAC/CRBL.txt.gz:BRAINEAC/FCTX.txt.gz:BRAINEAC/HIPP.txt.gz:BRAINEAC/MEDU.txt.gz:BRAINEAC/OCTX.txt.gz:BRAINEAC/PUTM.txt.gz:BRAINEAC/SNIG.txt.gz:BRAINEAC/TCTX.txt.gz:BRAINEAC/THAL.txt.gz:BRAINEAC/WHMT.txt.gz:BRAINEAC/aveALL.txt.gz:GTEx/v8/Adipose_Subcutaneous.txt.gz:GTEx/v8/Adipose_Visceral_Omentum.txt.gz:GTEx/v8/Adrenal_Gland.txt.gz:GTEx/v8/Cells_EBV-transformed_lymphocytes.txt.gz:GTEx/v8/Whole_Blood.txt.gz:GTEx/v8/Artery_Aorta.txt.gz:GTEx/v8/Artery_Coronary.txt.gz:GTEx/v8/Artery_Tibial.txt.gz:GTEx/v8/Brain_Amygdala.txt.gz:GTEx/v8/Brain_Anterior_cingulate_cortex_BA24.txt.gz:GTEx/v8/Brain_Caudate_basal_ganglia.txt.gz:GTEx/v8/Brain_Cerebellar_Hemisphere.txt.gz:GTEx/v8/Brain_Cerebellum.txt.gz:GTEx/v8/Brain_Cortex.txt.gz:GTEx/v8/Brain_Frontal_Cortex_BA9.txt.gz:GTEx/v8/Brain_Hippocampus.txt.gz:GTEx/v8/Brain_Hypothalamus.txt.gz:GTEx/v8/Brain_Nucleus_accumbens_basal_ganglia.txt.gz:GTEx/v8/Brain_Putamen_basal_ganglia.txt.gz:GTEx/v8/Brain_Spinal_cord_cervical_c-1.txt.gz:GTEx/v8/Brain_Substantia_nigra.txt.gz:GTEx/v8/Breast_Mammary_Tissue.txt.gz:GTEx/v8/Colon_Sigmoid.txt.gz:GTEx/v8/Colon_Transverse.txt.gz:GTEx/v8/Esophagus_Gastroesophageal_Junction.txt.gz:GTEx/v8/Esophagus_Mucosa.txt.gz:GTEx/v8/Esophagus_Muscularis.txt.gz:GTEx/v8/Heart_Atrial_Appendage.txt.gz:GTEx/v8/Heart_Left_Ventricle.txt.gz:GTEx/v8/Kidney_Cortex.txt.gz:GTEx/v8/Liver.txt.gz:GTEx/v8/Lung.txt.gz:GTEx/v8/Muscle_Skeletal.txt.gz:GTEx/v8/Nerve_Tibial.txt.gz:GTEx/v8/Ovary.txt.gz:GTEx/v8/Pancreas.txt.gz:GTEx/v8/Pituitary.txt.gz:GTEx/v8/Minor_Salivary_Gland.txt.gz:GTEx/v8/Cells_Cultured_fibroblasts.txt.gz:GTEx/v8/Skin_Not_Sun_Exposed_Suprapubic.txt.gz:GTEx/v8/Skin_Sun_Exposed_Lower_leg.txt.gz:GTEx/v8/Small_Intestine_Terminal_Ileum.txt.gz:GTEx/v8/Spleen.txt.gz:GTEx/v8/Stomach.txt.gz:GTEx/v8/Thyroid.txt.gz:GTEx/v8/Uterus.txt.gz:GTEx/v8/Vagina.txt.gz

eqtlMapSig = 1

eqtlMapP = 1

eqtlMapCADDth = 0

eqtlMapRDBth = NA

eqtlMapChr15 = NA

eqtlMapChr15Max = NA

eqtlMapChr15Meth = NA

eqtlMapAnnoDs = NA

eqtlMapAnnoMeth = NA

**[ciMap]**

ciMap = 1

ciMapBuiltin = EP/PsychENCODE/EP_links_oneway.txt.gz:HiC/PsychENCODE/Promoter_anchored_loops.txt.gz:EP/FANTOM5/EP_correlation_cell_type_oneway.txt.gz:EP/FANTOM5/EP_correlation_organ_oneway.txt.gz:HiC/Giusti-Rodriguez_et_al_2019/Adult_Cortex.txt.gz:HiC/Giusti-Rodriguez_et_al_2019/Fetal_Cortex.txt.gz:HiC/GSE87112/Adrenal.txt.gz:HiC/GSE87112/Aorta.txt.gz:HiC/GSE87112/Bladder.txt.gz:HiC/GSE87112/Dorsolateral_Prefrontal_Cortex.txt.gz:HiC/GSE87112/Hippocampus.txt.gz:HiC/GSE87112/Left_Ventricle.txt.gz:HiC/GSE87112/Liver.txt.gz:HiC/GSE87112/Lung.txt.gz:HiC/GSE87112/Ovary.txt.gz:HiC/GSE87112/Pancreas.txt.gz:HiC/GSE87112/Psoas.txt.gz:HiC/GSE87112/Right_Ventricle.txt.gz:HiC/GSE87112/Small_Bowel.txt.gz:HiC/GSE87112/Spleen.txt.gz:HiC/GSE87112/GM12878.txt.gz:HiC/GSE87112/IMR90.txt.gz:HiC/GSE87112/Mesenchymal_Stem_Cell.txt.gz:HiC/GSE87112/Mesendoderm.txt.gz:HiC/GSE87112/Neural_Progenitor_Cell.txt.gz:HiC/GSE87112/Trophoblast-like_Cell.txt.gz:HiC/GSE87112/hESC.txt.gz

ciMapFileN = 0

ciMapFiles = NA

ciMapFDR = 1e-6

ciMapPromWindow = 250-500

ciMapRoadmap = E080:E029:E030:E031:E032:E033:E034:E035:E036:E037:E038:E039:E040:E041:E042:E043:E044:E045:E046:E047:E048:E050:E051:E062:E053:E054:E067:E068:E069:E070:E071:E072:E073:E074:E081:E082:E027:E028:E001:E002:E003:E008:E014:E015:E016:E024:E004:E005:E006:E007:E009:E010:E011:E012:E013:E023:E025:E063:E075:E076:E106:E077:E078:E079:E084:E085:E109:E101:E102:E103:E092:E094:E110:E111:E083:E095:E104:E105:E086:E066:E017:E088:E096:E052:E089:E100:E107:E108:E090:E097:E087:E098:E091:E099:E055:E056:E057:E058:E059:E061:E113:E026:E049:E093:E112:E065:E018:E019:E020:E021:E022

ciMapEnhFilt = 0

ciMapPromFilt = 0

ciMapCADDth = 0

ciMapRDBth = NA

ciMapChr15 = NA

ciMapChr15Max = NA

ciMapChr15Meth = NA

ciMapAnnoDs = NA

ciMapAnnoMeth = NA

Note S2. Parameters for WebGestalt analyses

Enrichment method: ORA

Organism: hsapiens

Enrichment Categories: geneontology_Molecular_Function; geneontology_Bilogical_Process; geneontology_Cellular_Component

Interesting list: ENSG00000134982 ENSG00000012048 ENSG00000134852 ENSG00000116032 ENSG00000082701 ENSG00000226358 ENSG00000182333 ENSG00000204022 ENSG00000204021 ENSG00000204020 ENSG00000113580 ENSG00000142611 ENSG00000184719 ENSG00000256463 ENSG00000221955 ENSG00000196074 ENSG00000182087 ENSG00000129167 ENSG00000139287

Input ID type: ensembl_gene_id

Reference list: 61506 mapped entrezgene IDs from the platform genome (<http://www.webgestalt.org/api/reference?organism=hsapiens&referenceSet=genome>)

Minimum number of IDs in the category: 5

Maximum number of IDs in the category: 2000

FDR Method: BH

Significance Level: FDR < 0.05

Note S3. Parameters for g:profiler analyses

Version: e106_eg53_p16_65fcd97

Organism: hsapiens

Query length: 22

All results: false

Ordered: false

No iea: false

Sources: GO:MF, GO:CC, GO:BP

Multiquery: false

Numeric ns: ENTREZGENE_ACC

Domain: scope annotated

Measure underrepresentation: false

Significance threshold method: g_SCS

User threshold: 0.05

No evidences: false

Filter results: false

Input: ENSG00000134982 ENSG00000012048 ENSG00000134852 ENSG00000116032 ENSG00000082701 ENSG00000226358 ENSG00000182333 ENSG00000204022 ENSG00000204021 ENSG00000204020 ENSG00000113580 ENSG00000142611 ENSG00000184719 ENSG00000256463 ENSG00000221955 ENSG00000196074 ENSG00000182087 ENSG00000129167 ENSG00000139287

# Additional file Tables

Table S1. Characteristics of selected or not sub-cohort groups in cohort

| Variables | Not selected (N = 989) | Sub-cohort (N = 992) | P value |
| --- | --- | --- | --- |
| Age at baseline, years | 28.0(25.0-30.0) | 28.0(25.0-30.0) | 0.884^W^ |
| Baseline weight, kg | 55.0(50.0-61.0) | 56.0(51.0-62.0) | 0.072^W^ |
| Weight before pregnancy, kg | 53.0(48.0-59.3) | 54.0(49.0-60.0) | **0.016**^W^ |
| Height, cm | 160.0(158.0-163.0) | 160.0(158.0-164.0) | 0.380^W^ |
| Ethnic groups |  |  | 0.606 |
| Han | 976(99.4%) | 977(99.1%) |  |
| Minority | 6(0.6%) | 9(0.9%) |  |
| Education |  |  | 0.957 |
| Primary | 14(1.4%) | 17(1.7%) |  |
| Junior Secondary | 225(22.9%) | 222(22.5%) |  |
| Senior Secondary | 198(20.1%) | 196(19.9%) |  |
| College/university | 547(55.6%) | 551(55.9%) |  |
| Marriage |  |  | 0.237 |
| Divorced | 0(0.0%) | 1(0.1%) |  |
| Married | 953(96.6%) | 965(97.4%) |  |
| Unmarried | 34(3.4%) | 25(2.5%) |  |
| Family annual income, RMB |  |  | 0.480 |
| Less than 100000 | 325(40.7%) | 300(37.7%) |  |
| 100000 - 200000 | 394(49.3%) | 398(50.0%) |  |
| 200000 - 300000 | 58(7.3%) | 75(9.4%) |  |
| 300000 - 400000 | 9(1.1%) | 13(1.6%) |  |
| 400000 - 500000 | 8(1.0%) | 7(0.9%) |  |
| More than 500000 | 5(0.6%) | 3(0.4%) |  |
| Family history |  |  |  |
| Diabetes mellitus |  |  | 0.892 |
| No | 928(94.0%) | 920(92.7%) |  |
| Yes | 59(6.0%) | 72(7.3%) |  |
| Hypertension |  |  | 0.526 |
| No | 811(82.2%) | 815(82.2%) |  |
| Yes | 176(17.8%) | 177(17.8%) |  |
| Age of menarche | 14.0(13.0-15.0) | 14.0(13.0-15.0) | 0.237 |
| Menstrual conditions |  |  | 0.406 |
| No | 168(17.1%) | 173(17.6%) |  |
| Yes | 817(82.9%) | 812(82.4%) |  |
| GA at baseline, weeks | 16.4(15.9-17.1) | 16.4(15.9-17.1) | 0.608^W^ |
| GA ending pregnancy, weeks | 39.3(38.6-40.0) | 39.3(38.6-40.1) | 0.684^W^ |
| Primiparity |  |  | 0.881 |
| No | 575(58.4%) | 554(55.8%) |  |
| Yes | 410(41.6%) | 438(44.2%) |  |
| IVF pregnancy |  |  | 0.878 |
| No | 968(98.9%) | 963(98.4%) |  |
| Yes | 11(1.1%) | 16(1.6%) |  |
| Active smoking |  |  | 0.058 |
| Ceased after pregnancy | 15(1.5%) | 29(2.9%) |  |
| Current smoking | 1(0.1%) | 2(0.2%) |  |
| Never | 966(98.4%) | 953(96.8%) |  |
| Alcohol drinking |  |  | 0.695 |
| Ceased after pregnancy | 151(15.4%) | 166(16.9%) |  |
| Current drinking | 1(0.1%) | 1(0.1%) |  |
| Never | 828(84.5%) | 815(83.0%) |  |
| Passive smoking every week |  |  | 0.543 |
| 0 day | 614(79.2%) | 610(77.0%) |  |
| 1 - 3 days | 77(9.9%) | 96(12.1%) |  |
| 4 - 5 days | 18(2.3%) | 21(2.7%) |  |
| 6 - 7 days | 66(8.5%) | 65(8.2%) |  |
| Medical record of delivery |  |  | 0.543 |
| No | 69(7.0%) | 69(7.0%) |  |
| Yes | 920(93.0%) | 923(93.0%) |  |
| Genotyping data |  |  | 0.795 |
| No | 89(9.0%) | 80(8.1%) |  |
| Yes | 900(91.0%) | 912(91.9%) |  |

Values are n (%), or median (interquartile range). P value less than 0.05 was bold. P values with superscript “W” were calculated by Wilcoxon signed-rank test, and others were calculated by Fisher's exact test. There is missing data for some variables.

GA = gestational age; IVF = in-vitro fertilization.

Table S2. PCs with P value < 0.01 for SNPs/InDels association analyses

| **PC** | **Eigenvalue** | **Difference** | **Twstat** | **P value** | **Effect.n** |
| --- | --- | --- | --- | --- | --- |
| 1 | 1.32 | NA | 36.20 | 4.9e-65 | 187494.63 |
| 2 | 1.26 | -0.062 | 4.12 | 1.7e-04 | 321477.31 |
| 3 | 1.25 | -0.004 | 2.78 | 2.6e-03 | 345779.60 |

InDel = insertions-deletion; NA = Not applicable; PC = Principle component; SNP = Single nucleotide polymorphism.

Table S3. Markers in linkage disequilibrium with any independent significant markers (r^2^ ≥ 0.6)

| **rsID** | **Chr** | **Pos** | **Minor Allele** | **Major Allele** | **MAF** | **IndSigMark** | **Genomic**  **Locus** | **Nearest Gene** | | |
| --- | --- | --- | --- | --- | --- | --- | --- | --- | --- | --- |
|  |  |  |  |  |  |  |  | **Symbol** | **Distance** | **Function** |
| rs2948819 | 3 | 124799357 | T | C | 0.13 | rs78175392 | 1 | SLC12A8 | 2122 | Intergenic |
| rs2981488 | 3 | 124800082 | G | A | 0.13 | rs78175392 | 1 | SLC12A8 | 1397 | Intergenic |
| rs558738524 | 3 | 124800851 | CT | C | 0.15 | rs78175392 | 1 | SLC12A8 | 628 | Downstream |
| rs75686093 | 3 | 124802914 | G | C | 0.08 | rs78175392 | 1 | SLC12A8 | 0 | Intronic |
| rs2981477 | 3 | 124804239 | A | T | 0.15 | rs78175392 | 1 | SLC12A8 | 0 | Intronic |
| rs78175392 | 3 | 124808719 | T | C | 0.11 | rs78175392 | 1 | SLC12A8 | 0 | Intronic |
| rs2948808 | 3 | 124809172 | C | T | 0.15 | rs78175392 | 1 | SLC12A8 | 0 | Intronic |
| rs2333042 | 3 | 124812169 | C | T | 0.10 | rs78175392 | 1 | SLC12A8 | 0 | Intronic |
| rs3965994 | 3 | 124812328 | T | G | 0.10 | rs78175392 | 1 | SLC12A8 | 0 | Intronic |
| rs75686195 | 3 | 124814875 | A | G | 0.09 | rs78175392 | 1 | SLC12A8 | 0 | Intronic |
| rs4679346 | 3 | 124817984 | C | G | 0.09 | rs78175392 | 1 | SLC12A8 | 0 | Intronic |
| rs57686780 | 10 | 90422492 | A | C | 0.47 | rs12253503 | 2 | LIPF | 1705 | Intergenic |
| rs201003503 | 10 | 90432382 | AACACACACACAC | A | 0.38 | rs12253503 | 2 | LIPF | 0 | Intronic |
| rs4333926 | 10 | 90433994 | C | G | 0.49 | rs12253503 | 2 | LIPF | 0 | Intronic |
| rs3858282 | 10 | 90434411 | T | C | 0.49 | rs12253503 | 2 | LIPF | 0 | Intronic |
| rs11202811 | 10 | 90446873 | A | T | 0.49 | rs12253503 | 2 | LIPF | 8301 | Intergenic |
| rs6586149 | 10 | 90449236 | A | G | 0.43 | rs12253503 | 2 | RP11-186O14.7 | 10203 | Intergenic |
| rs7080521 | 10 | 90450498 | A | T | 0.50 | rs12253503 | 2 | RP11-186O14.7 | 8941 | Intergenic |
| rs61296034 | 10 | 90452165 | T | G | 0.47 | rs12253503 | 2 | RP11-186O14.7 | 7274 | Intergenic |
| rs1469631 | 10 | 90452938 | T | C | 0.48 | rs12253503 | 2 | RP11-186O14.7 | 6501 | Intergenic |
| rs1469632 | 10 | 90453089 | C | G | 0.48 | rs12253503 | 2 | RP11-186O14.7 | 6350 | Intergenic |
| rs5786831 | 10 | 90454684 | TTA | T | 0.48 | rs12253503 | 2 | RP11-186O14.7 | 4755 | Intergenic |
| rs80203957 | 10 | 90454773 | A | C | 0.43 | rs12253503 | 2 | RP11-186O14.7 | 4666 | Intergenic |
| rs201359564 | 10 | 90455363 | TAC | T | 0.16 | rs12253503 | 2 | RP11-186O14.7 | 4076 | Na |
| rs1331063 | 10 | 90455363 | C | T | 0.48 | rs12253503 | 2 | RP11-186O14.7 | 4076 | Intergenic |
| rs11202818 | 10 | 90456506 | T | C | 0.49 | rs12253503 | 2 | RP11-186O14.7 | 2933 | Intergenic |
| rs11202819 | 10 | 90456564 | C | G | 0.44 | rs12253503 | 2 | RP11-186O14.7 | 2875 | Intergenic |
| rs1368180 | 10 | 90457129 | A | T | 0.49 | rs12253503 | 2 | RP11-186O14.7 | 2310 | Intergenic |
| rs3068333 | 10 | 90457385 | A | ATC | 0.44 | rs12253503 | 2 | RP11-186O14.7 | 2054 | Intergenic |
| rs2862082 | 10 | 90457389 | T | G | 0.44 | rs12253503 | 2 | RP11-186O14.7 | 2050 | Intergenic |
| rs1888606 | 10 | 90457633 | C | A | 0.45 | rs12253503 | 2 | RP11-186O14.7 | 1806 | Intergenic |
| rs1549857 | 10 | 90459810 | G | A | 0.48 | rs12253503 | 2 | RP11-186O14.7 | 0 | Ncrna_exonic |
| rs3928442 | 10 | 90459826 | A | G | 0.48 | rs12253503 | 2 | RP11-186O14.7 | 0 | Ncrna_exonic |
| rs139080864 | 10 | 90461348 | ACTT | A | 0.48 | rs12253503 | 2 | RP11-186O14.7 | 1189 | Intergenic |
| rs12246792 | 10 | 90461415 | G | A | 0.49 | rs12253503 | 2 | RP11-186O14.7 | 1256 | Intergenic |
| rs12253503 | 10 | 90461441 | A | G | 0.48 | rs12253503 | 2 | RP11-186O14.7 | 1282 | Intergenic |
| rs12266745 | 10 | 90461634 | T | A | 0.49 | rs12253503 | 2 | RP11-186O14.7 | 1475 | Intergenic |
| rs72634989 | 10 | 90461823 | C | T | 0.49 | rs12253503 | 2 | RP11-186O14.7 | 1664 | Intergenic |
| rs12267931 | 10 | 90466296 | C | T | 0.43 | rs12253503 | 2 | RP11-186O14.7 | 6137 | Intergenic |

Chr = chromosome; IndSigMark = Independent significant marker; MAF = minor allele frequency computed based on the reference panel; Pos = position on hg19.

Table S4. Genes mapped by positional mapping, eQTL mapping, and chromatin interaction mapping

| **Gene** | **ENSGID** | **Chr** | **Start** | **End** | **Type** | **PosMap** | **eqtlMap** | **ciMap** | **IndSigMark** |
| --- | --- | --- | --- | --- | --- | --- | --- | --- | --- |
| RP11-71H17.7 | ENSG00000260391 | 3 | 124442635 | 124445172 | sense_overlapping | N | N | Y | rs78175392 |
| UMPS | ENSG00000114491 | 3 | 124449213 | 124464040 | protein_coding | N | N | Y | rs78175392 |
| MIR544B | ENSG00000265981 | 3 | 124451286 | 124451363 | miRNA | N | N | Y | rs78175392 |
| RP11-71H17.1 | ENSG00000242199 | 3 | 124452265 | 124452601 | pseudogene | N | N | Y | rs78175392 |
| RP11-71H17.9 | ENSG00000272947 | 3 | 124467422 | 124468120 | lincRNA | N | N | Y | rs78175392 |
| ITGB5-AS1 | ENSG00000244286 | 3 | 124500002 | 124506604 | antisense | N | N | Y | rs78175392 |
| ENO1P3 | ENSG00000243986 | 3 | 124580941 | 124582164 | pseudogene | N | N | Y | rs78175392 |
| MUC13 | ENSG00000173702 | 3 | 124624289 | 124672663 | protein_coding | N | N | Y | rs78175392 |
| HEG1 | ENSG00000173706 | 3 | 124684554 | 124774802 | protein_coding | N | N | Y | rs78175392 |
| RNA5SP137 | ENSG00000252642 | 3 | 124777148 | 124777282 | rRNA | N | N | Y | rs78175392 |
| SLC12A8 | ENSG00000221955 | 3 | 124801480 | 124998021 | protein_coding | Y | Y | Y | rs78175392 |
| RNU6-230P | ENSG00000199327 | 3 | 124837941 | 124838044 | snRNA | N | N | Y | rs78175392 |
| MIR5092 | ENSG00000264986 | 3 | 124870309 | 124870396 | miRNA | N | N | Y | rs78175392 |
| ZNF148 | ENSG00000163848 | 3 | 124944405 | 125094198 | protein_coding | N | N | Y | rs78175392 |
| DUTP1 | ENSG00000229048 | 3 | 125029725 | 125030194 | pseudogene | N | N | Y | rs78175392 |
| RP11-775J23.2 | ENSG00000243508 | 3 | 125062929 | 125063641 | pseudogene | N | N | Y | rs78175392 |
| RNU6-232P | ENSG00000252953 | 3 | 125107778 | 125107841 | snRNA | N | N | Y | rs78175392 |
| snoU13 | ENSG00000238992 | 3 | 125164905 | 125165007 | snoRNA | N | N | Y | rs78175392 |
| SNX4 | ENSG00000114520 | 3 | 125165495 | 125239041 | protein_coding | N | N | Y | rs78175392 |
| Y_RNA | ENSG00000201800 | 3 | 125235823 | 125235930 | misc_RNA | N | N | Y | rs78175392 |
| Y_RNA | ENSG00000252965 | 3 | 125247088 | 125247193 | misc_RNA | N | N | Y | rs78175392 |
| OSBPL11 | ENSG00000144909 | 3 | 125247702 | 125313934 | protein_coding | N | N | Y | rs78175392 |
| OR7E29P | ENSG00000243429 | 3 | 125430983 | 125431889 | pseudogene | N | N | Y | rs78175392 |
| ALG1L | ENSG00000189366 | 3 | 125648118 | 125655882 | protein_coding | N | Y | N | rs78175392 |
| SLC41A3 | ENSG00000114544 | 3 | 125725198 | 125820404 | protein_coding | N | Y | N | rs78175392 |
| Y_RNA | ENSG00000222192 | 10 | 89322845 | 89322943 | misc_RNA | N | N | Y | rs12253503 |
| RP11-57C13.4 | ENSG00000223761 | 10 | 89351364 | 89365781 | lincRNA | N | N | Y | rs12253503 |
| RP11-57C13.6 | ENSG00000225913 | 10 | 89367742 | 89419036 | lincRNA | N | N | Y | rs12253503 |
| RP11-57C13.3 | ENSG00000196566 | 10 | 89369920 | 89419760 | antisense | N | N | Y | rs12253503 |
| RP11-57C13.5 | ENSG00000234192 | 10 | 89402364 | 89402711 | pseudogene | N | N | Y | rs12253503 |
| PAPSS2 | ENSG00000198682 | 10 | 89419370 | 89507462 | protein_coding | N | N | Y | rs12253503 |
| ATAD1 | ENSG00000138138 | 10 | 89511269 | 89601100 | protein_coding | N | N | Y | rs12253503 |
| CFL1P1 | ENSG00000223820 | 10 | 89577685 | 89605369 | pseudogene | N | N | Y | rs12253503 |
| RN7SL78P | ENSG00000243782 | 10 | 89592578 | 89592855 | misc_RNA | N | N | Y | rs12253503 |
| KLLN | ENSG00000227268 | 10 | 89618918 | 89623194 | protein_coding | N | N | Y | rs12253503 |
| PTEN | ENSG00000171862 | 10 | 89622870 | 89731687 | protein_coding | N | N | Y | rs12253503 |
| RP11-380G5.2 | ENSG00000224745 | 10 | 89638449 | 89640184 | sense_intronic | N | N | Y | rs12253503 |
| RP11-380G5.3 | ENSG00000213613 | 10 | 89705259 | 89705781 | pseudogene | N | N | Y | rs12253503 |
| SNORD74 | ENSG00000200891 | 10 | 89754375 | 89754452 | snoRNA | N | N | Y | rs12253503 |
| MED6P1 | ENSG00000227905 | 10 | 89807892 | 89809580 | pseudogene | N | N | Y | rs12253503 |
| RNLS | ENSG00000184719 | 10 | 90033621 | 90344287 | protein_coding | N | Y | Y | rs12253503 |
| Y_RNA | ENSG00000201548 | 10 | 90345395 | 90345490 | misc_RNA | N | N | Y | rs12253503 |
| LIPJ | ENSG00000204022 | 10 | 90346510 | 90366733 | protein_coding | N | Y | Y | rs12253503 |
| RPL7P34 | ENSG00000237205 | 10 | 90377980 | 90378691 | pseudogene | N | N | Y | rs12253503 |
| LIPF | ENSG00000182333 | 10 | 90424198 | 90438571 | protein_coding | Y | N | Y | rs12253503 |
| RP11-186O14.7 | ENSG00000271408 | 10 | 90459440 | 90460158 | pseudogene | Y | N | Y | rs12253503 |
| LIPK | ENSG00000204021 | 10 | 90484301 | 90512543 | protein_coding | N | Y | Y | rs12253503 |
| KRT8P38 | ENSG00000226358 | 10 | 90487242 | 90488671 | pseudogene | N | Y | Y | rs12253503 |
| LIPN | ENSG00000204020 | 10 | 90521163 | 90537999 | protein_coding | N | Y | Y | rs12253503 |
| RCBTB2P1 | ENSG00000235150 | 10 | 90545357 | 90546818 | pseudogene | N | N | Y | rs12253503 |
| LIPM | ENSG00000173239 | 10 | 90562487 | 90580303 | protein_coding | N | N | Y | rs12253503 |
| ANKRD22 | ENSG00000152766 | 10 | 90581889 | 90611575 | protein_coding | N | N | Y | rs12253503 |
| PTCD2P2 | ENSG00000234256 | 10 | 90636207 | 90637301 | pseudogene | N | N | Y | rs12253503 |
| STAMBPL1 | ENSG00000138134 | 10 | 90639491 | 90734910 | protein_coding | N | N | Y | rs12253503 |
| ACTA2-AS1 | ENSG00000180139 | 10 | 90692400 | 90700368 | antisense | N | N | Y | rs12253503 |
| ACTA2 | ENSG00000107796 | 10 | 90694831 | 90751147 | protein_coding | N | Y | N | rs12253503 |
| RP11-399O19.9 | ENSG00000261438 | 10 | 90775593 | 90776816 | sense_overlapping | N | N | Y | rs12253503 |
| snoU13 | ENSG00000238991 | 10 | 90820788 | 90820888 | snoRNA | N | N | Y | rs12253503 |
| MIR4679-2 | ENSG00000265375 | 10 | 90823092 | 90823168 | miRNA | N | N | Y | rs12253503 |
| RP11-341B24.3 | ENSG00000233292 | 10 | 90898801 | 90899215 | pseudogene | N | N | Y | rs12253503 |
| CH25H | ENSG00000138135 | 10 | 90965694 | 90967071 | protein_coding | N | N | Y | rs12253503 |
| RP11-168O10.6 | ENSG00000234452 | 10 | 91215821 | 91227897 | antisense | N | N | Y | rs12253503 |
| RP11-478K7.2 | ENSG00000226159 | 10 | 91675246 | 91717130 | lincRNA | N | N | Y | rs12253503 |
| SNRPD2P1 | ENSG00000228938 | 10 | 91738474 | 91738827 | pseudogene | N | N | Y | rs12253503 |

Chr = chromosome; ciMap = Chromatin interaction mapping; eqtlMap = eQTL mapping; IndSigMark = Independent significant marker; N = not mapped; PosMap = Positional mapping; Y = mapped.

Table S5. Protein coding genes from MAGMA gene-based analyses with P_Bon_ < 0.05

| **Gene** | **Chr** | **Start** | **Stop** | **P** | **P_Bon_** |
| --- | --- | --- | --- | --- | --- |
| ERBB4 | 2 | 212240446 | 213403565 | 5.7e-07 | 0.0006 |
| SLC12A8 | 3 | 124801480 | 124998021 | 3.5e-06 | 0.0035 |
| TENM4 | 11 | 78363876 | 79151992 | 3.6e-06 | 0.0036 |
| NCKAP5 | 2 | 133429374 | 134326034 | 3.6e-06 | 0.0036 |
| IL1R1 | 2 | 102681004 | 102796334 | 5.3e-06 | 0.0054 |
| DMD | X | 31115794 | 33357558 | 8.1e-06 | 0.0082 |
| LDB2 | 4 | 16503164 | 16900432 | 1.1e-05 | 0.0109 |
| PCSK5 | 9 | 78505560 | 78977255 | 1.8e-05 | 0.0178 |
| ARHGAP26 | 5 | 142149949 | 142608576 | 2.0e-05 | 0.0204 |
| KCNE1 | 21 | 35818988 | 35884573 | 2.5e-05 | 0.0253 |
| CCDC85C | 14 | 99977603 | 100070363 | 2.7e-05 | 0.0271 |
| ANO10 | 3 | 43396351 | 43733086 | 4.0e-05 | 0.0405 |

Chr = chromosome; PBon = Bonferroni corrected P value. The number of mapped protein coding genes is 1007.

Table S6. CNVRs associated with GDM FDR with p value < 0.05

| **CNVR_ID** | **Chr** | **Start** | **End** | **CNVRType** | **Gene** | **Envents in cases**  **N (%)** | **Envents in controls**  **N (%)** | **RR(95CI)** | **P value** | | |
| --- | --- | --- | --- | --- | --- | --- | --- | --- | --- | --- | --- |
|  |  |  |  |  |  |  |  |  | P | Bonferroni | FDR |
| CNVR_10_1 | 1 | 3328124 | 3331193 | Mixed | PRDM16 | 23 (11.9) | 32 (3.9) | 2.2 (1.6-3.2) | 9.70e-06 | 2.10e-03 | 2.10e-03 |
| CNVR_2324_1 | 18 | 76715856 | 76769285 | Mixed | SALL3 | 28 (14.5) | 51 (6.2) | 2.0 (1.4-2.7) | 2.00e-05 | 4.30e-03 | 2.10e-03 |
| CNVR_824_3 | 5 | 142727129 | 142779074 | Mixed | NR3C1 | 30 (15.5) | 76 (9.3) | 1.9 (1.4-2.5) | 3.50e-05 | 7.70e-03 | 2.60e-03 |
| CNVR_2494_1 | 20 | 58437036 | 58502456 | Mixed | SYCP2 | 53 (27.5) | 112 (13.7) | 1.7 (1.3-2.3) | 5.00e-05 | 1.10e-02 | 2.80e-03 |
| CNVR_2222_1 | 17 | 41228500 | 41228632 | Mixed | BRCA1 | 23 (11.9) | 34 (4.2) | 2.0 (1.4-2.9) | 1.90e-04 | 4.10e-02 | 8.20e-03 |
| CNVR_290_1 | 2 | 89830707 | 89873480 | Mixed | NA | 21 (10.9) | 211 (25.8) | 0.5 (0.3-0.7) | 3.70e-04 | 8.20e-02 | 1.20e-02 |
| CNVR_2223_1 | 17 | 41244188 | 41244217 | Mixed | BRCA1 | 42 (21.8) | 76 (9.3) | 1.8 (1.3-2.5) | 4.20e-04 | 9.30e-02 | 1.20e-02 |
| CNVR_631_4 | 4 | 46433505 | 46477217 | Mixed | NA | 20 (10.4) | 59 (7.2) | 1.8 (1.3-2.6) | 4.50e-04 | 1.00e-01 | 1.20e-02 |
| CNVR_2332_1 | 19 | 1000440 | 1016849 | Mixed | GRIN3B, TMEM259 | 62 (32.1) | 253 (30.9) | 1.5 (1.2-2.0) | 1.10e-03 | 2.30e-01 | 2.60e-02 |
| CNVR_799_1 | 5 | 112111323 | 112112584 | Mixed | APC | 44 (22.8) | 97 (11.8) | 1.6 (1.2-2.2) | 1.50e-03 | 3.30e-01 | 3.10e-02 |
| CNVR_1736_1 | 12 | 72335073 | 72336769 | Gain | TPH2 | 15 (7.8) | 42 (5.1) | 1.8 (1.3-2.7) | 1.60e-03 | 3.40e-01 | 3.10e-02 |
| CNVR_1552_1 | 11 | 18039772 | 18060900 | Gain | TPH1 | 41 (21.2) | 137 (16.7) | 1.5 (1.2-1.9) | 2.00e-03 | 4.30e-01 | 3.60e-02 |
| CNVR_635_1 | 4 | 56328468 | 56372665 | Gain | CLOCK | 23 (11.9) | 75 (9.2) | 1.6 (1.2-2.2) | 2.20e-03 | 4.90e-01 | 3.80e-02 |
| CNVR_516_1 | 3 | 119680558 | 119716098 | Mixed | GSK3B | 32 (16.6) | 103 (12.6) | 1.6 (1.2-2.1) | 2.80e-03 | 6.20e-01 | 4.40e-02 |

Chr = chromosome; CI = Confidence interval; CNVR = copy number variant region; FDR = False discovery rate; GDM = gestational diabetes mellitus; RR = Risk ratio. The number of tested CNVRs is 220.

Table S7. Candidate genes for gene over-representation analyses

| **Gene** | **Gene name** | **NCBI link** | **Genecards link** |
| --- | --- | --- | --- |
| APC | APC, WNT signaling pathway regulator | <https://www.ncbi.nlm.nih.gov/gene/?term=324> | <https://www.genecards.org/cgi-bin/carddisp.pl?gene=APC> |
| BRCA1 | BRCA1, DNA repair associated | <https://www.ncbi.nlm.nih.gov/gene/?term=672> | <https://www.genecards.org/cgi-bin/carddisp.pl?gene=BRCA1> |
| CLOCK | Clock circadian regulator | <https://www.ncbi.nlm.nih.gov/gene/?term=9575> | <https://www.genecards.org/cgi-bin/carddisp.pl?gene=CLOCK> |
| GRIN3B | Glutamate ionotropic receptor nmda type subunit 3b | <https://www.ncbi.nlm.nih.gov/gene/?term=116444> | <https://www.genecards.org/cgi-bin/carddisp.pl?gene=GRIN3B> |
| GSK3B | Glycogen synthase kinase 3 beta | <https://www.ncbi.nlm.nih.gov/gene/?term=2932> | <https://www.genecards.org/cgi-bin/carddisp.pl?gene=GSK3B> |
| KRT8P38 | Keratin 8 pseudogene 38 | <https://www.ncbi.nlm.nih.gov/gene/?term=100418799> | <https://www.genecards.org/cgi-bin/carddisp.pl?gene=KRT8P38> |
| LIPF | Lipase F, gastric type | <https://www.ncbi.nlm.nih.gov/gene/?term=8513> | <https://www.genecards.org/cgi-bin/carddisp.pl?gene=LIPF> |
| LIPJ | Lipase family member J | <https://www.ncbi.nlm.nih.gov/gene/?term=142910> | <https://www.genecards.org/cgi-bin/carddisp.pl?gene=LIPJ> |
| LIPK | Lipase family member K | <https://www.ncbi.nlm.nih.gov/gene/?term=643414> | <https://www.genecards.org/cgi-bin/carddisp.pl?gene=LIPK> |
| LIPN | Lipase family member N | <https://www.ncbi.nlm.nih.gov/gene/?term=643418> | <https://www.genecards.org/cgi-bin/carddisp.pl?gene=LIPN> |
| NR3C1 | Nuclear receptor subfamily 3 group C member 1 | <https://www.ncbi.nlm.nih.gov/gene/?term=2908> | <https://www.genecards.org/cgi-bin/carddisp.pl?gene=NR3C1> |
| PRDM16 | PR/SET domain 16 | <https://www.ncbi.nlm.nih.gov/gene/?term=63976> | <https://www.genecards.org/cgi-bin/carddisp.pl?gene=PRDM16> |
| RNLS | Renalase, FAD dependent amine oxidase | <https://www.ncbi.nlm.nih.gov/gene/?term=55328> | <https://www.genecards.org/cgi-bin/carddisp.pl?gene=RNLS> |
| SALL3 | Spalt like transcription factor 3 | <https://www.ncbi.nlm.nih.gov/gene/?term=27164> | <https://www.genecards.org/cgi-bin/carddisp.pl?gene=SALL3> |
| SLC12A8 | Solute carrier family 12 member 8 | <https://www.ncbi.nlm.nih.gov/gene/?term=84561> | <https://www.genecards.org/cgi-bin/carddisp.pl?gene=SLC12A8> |
| SYCP2 | Synaptonemal complex protein 2 | <https://www.ncbi.nlm.nih.gov/gene/?term=10388> | <https://www.genecards.org/cgi-bin/carddisp.pl?gene=SYCP2> |
| TMEM259 | Transmembrane protein 259 | <https://www.ncbi.nlm.nih.gov/gene/?term=91304> | <https://www.genecards.org/cgi-bin/carddisp.pl?gene=TMEM259> |
| TPH1 | Tryptophan hydroxylase 1 | <https://www.ncbi.nlm.nih.gov/gene/?term=7166> | <https://www.genecards.org/cgi-bin/carddisp.pl?gene=TPH1> |
| TPH2 | Tryptophan hydroxylase 2 | <https://www.ncbi.nlm.nih.gov/gene/?term=121278> | <https://www.genecards.org/cgi-bin/carddisp.pl?gene=TPH2> |

Table S8. Gene sets enriched in the Gene Ontology resource by g: Profiler

| **Gene set** | **Description** | **Ontology** | **Gene** | **P_adj_** | **Definition and comment** |
| --- | --- | --- | --- | --- | --- |
| GO:0004806 | Triglyceride lipase activity | MF | LIPF, LIPK, LIPN | 0.000 | Catalysis of the hydrolysis of triglyceride to diglyceride. |
| GO:0016298 | Lipase activity | MF | LIPF, LIPJ, LIPK, LIPN | 0.048 | Catalysis of the hydrolysis of a lipid or phospholipid. |
| GO:0004465 | Lipoprotein lipase activity | MF | LIPK, LIPN | 0.008 | Catalysis of the hydrolysis of Triglyceride in lipoprotein to diglyceride. |
| GO:0016714 | Oxidoreductase activity | MF | TPH1, TPH2 | 0.004 | Catalysis of an oxidation-reduction reaction. |
| GO:0004510 | Tryptophan 5-monooxygenase activity | MF | TPH1, TPH2 | 0.000 | Catalysis of the hydroxylase of L-tryptophan to 5-hydroxy-L-tryptophan, the primary product that converts l-tryptophan into 5-hydroxytryptamine. |
| GO:0030877 | Beta-catenin destruction complex | CC | APC, GSK3B | 0.008 | A cytoplasmic protein complex containing glycogen synthase kinase-3-beta (GSK3-beta). |
| GO:1990909 | Wnt signalosome | CC | APC, GSK3B | 0.008 | A multiprotein protein complex containing membrane-localized Wnt receptors and cytosolic protein complexes. Within the Wnt signalosome, beta-catenin phosphorylation is inhibited through internalization of the signalosome complex, resulting in the sequestration of GSK3-beta into multi-vesicular bodies. No longer phosphorylated and targeted for degradation, beta-catenin is free to enter the nucleus and modulate downstream transcription. |
| GO:0000800 | Lateral element | CC | BRCA1, SYCP2 | 0.011 | A proteinaceous core found between sister chromatids during meiotic prophase. They are part of the synaptonemal complex. |
| GO:0042427 | Serotonin biosynthetic process | BP | TPH1, TPH2 | 0.007 | The chemical reactions and pathways resulting in the formation of 5-hydroxytryptamine (serotonin), a monoamine neurotransmitter occurring in the peripheral and central nervous systems, also having hormonal properties. |
| GO:0042435 | Indole-containing compound biosynthetic process | BP | TPH1, TPH2 | 0.017 | The chemical reactions and pathways resulting in the formation of compounds that contain an indole (2,3-benzopyrrole) skeleton. |
| GO:1901162 | Primary amino compound biosynthetic process | BP | TPH1, TPH2 | 0.017 | The chemical reactions and pathways resulting in the formation of primary amino compound. |
| GO:1904781 | Positive regulation of protein localization to centrosome | BP | APC, GSK3B | 0.023 | Any process that activates or increases the frequency, rate, or extent of protein localization to centrosome. |
| GO:0070268 | Cornification | BP | LIPK, LIPN | 0.031 | A type of programmed cell death that occurs in the epidermis. |
| GO:2000074 | Regulation of type B pancreatic cell development | BP | CLOCK, GSK3B | 0.040 | Any process that modulates the frequency, rate, or extent of pancreatic B cell development. |
| GO:0007623 | Circadian rhythm | BP | CLOCK, GSK3B, TPH1, TPH2 | 0.045 | Any biological process in an organism that recurs with a regularity of approximately 24 hours. |
| GO:1904779 | Regulation of protein localization to centrosome | BP | APC, GSK3B | 0.050 | Any process that modulates the frequency, rate, or extent of protein localization to centrosome. |

GO = Gene Ontology; P_FDR_ = P value corrected by false discovery rate method in WebGestalt analysis. P_adj_ = P value corrected by g:SCS method which corresponds to an experiment-wide threshold of α = 0.05 in g:Profiler analysis.

BP = Biological process; CC = Cellular component; MF = Molecular function.

Table S9. Genome-wide association studies of GDM reported in literatures and the present study

| **Study** | **Type** | **Site** | **Subjects** | **Sample size** | **GDM diagnosis** | **Genotyping chip** | **Imputation** | **Variant** | **Sig. threshold** | **Sig. marker mapped with gene** | |
| --- | --- | --- | --- | --- | --- | --- | --- | --- | --- | --- | --- |
|  |  |  |  |  |  |  |  |  |  | **Marker** | **Nearest gene** |
| Kwak et al. | Case control study | Korea | GDM case:  Cheil General Hospital;  Control:  age ≥ 50 years;  no history of T2DM;  no first-degree relatives with T2DM;  fasting plasma glucose < 5.6 mmol/L, and HbA1c < 6.0%;  No information GDM history for controls. | GDM: 468  Control: 1242 | 100 g OGTT (≥ 2 terms):  fasting ≥ 5.8 mmol/L  1 h ≥ 10.6 mmol/L  2 h ≥ 9.2 mmol/L  3 h ≥ 8.1 mmol/L | Affymetrix Genome-Wide Human SNP Array 5.0 | Software: IMPUTE V1  Reference: HapMap-phased genotype information of Japanese in  Tokyo, Japan, and Han Chinese in Beijing, China (build 36 release 21) | SNP | 2e-5 | rs7754840  rs10830962  rs1470579  rs6499500  rs12898654  rs9395950  rs7513574  rs12715106  rs187230 | CDKAL1  MTNR1B  IGF2BP2  FTSJD1/CALB2  LBXCOR1  TINAG  CACHD  LRRC3B  PLD1 |
| Wu et al. | Case control study | Beijing: Luhe hospital,  Tongzhou Maternal & Child Health  Hospital | Chinese Han;  pregnant;  19-44 years old;  no family history of diabetes for controls. | GDM: 103  Control: 115 | 75 g OGTT (≥ 1 terms):  fasting 5.1-6.9 mmol/L  1 h ≥ 10.0 mmol/L  2 h 8.5-11.0 mmol/L | Illumina Human OmniZhongHua-8 Bead Chip | No | SNP | 1e-4 | rs12491231  kgp12506180  kgp3680296  rs1347523  rs4939551  rs476496 | SYNPR  CDH18  CDH18  CDH18  CTIF  PTGIS |
| This study | Case cohort study | Anqing Municipal Hospital, Anhui Province | Please see the criteria in Methods. | GDM: 193  Control: 819 | Please see in Methods. | Infinium Asian Screening Array-China Health Industry Alliance Bead chip | Software: IMPUTE V2  Reference: 1000 Genomes Project phase 3 East Asian population | SNP  InDel  CNV | **SNP/InDel:**  1e-5  **CNV:**  false discovery rate corrected p value < 0.05 | **SNP:**  rs78175392  rs12253503 | **SNP:**  SLC12A8  RP11-186O14.7  **CNV:**  APC  BRCA1  CLOCK  GRIN3B  GSK3B  NR3C1  PRDM16  SALL3  SLC12A8  SYCP2  TMEM259  TPH1  TPH2 |

# Additional file Figures


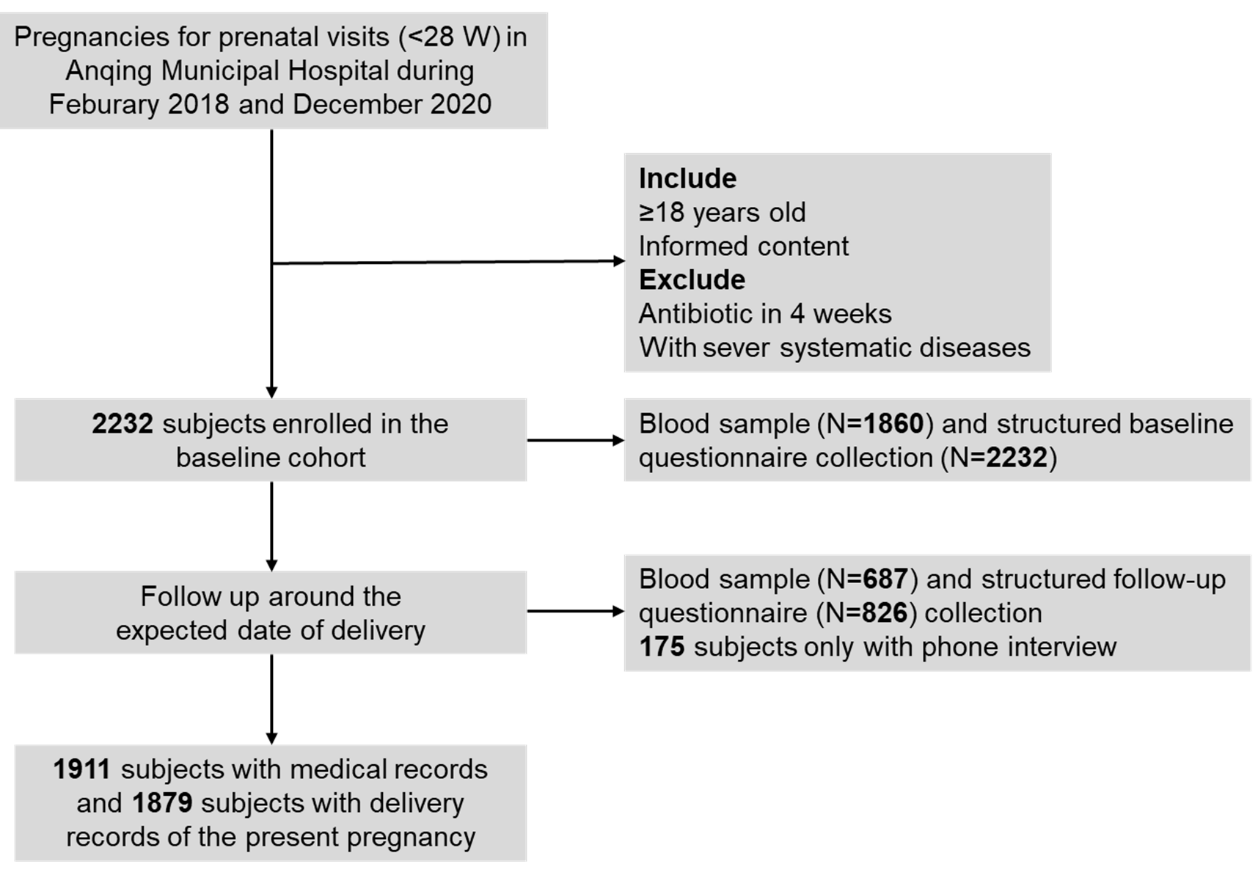


Figure S1. Profile of the study cohort


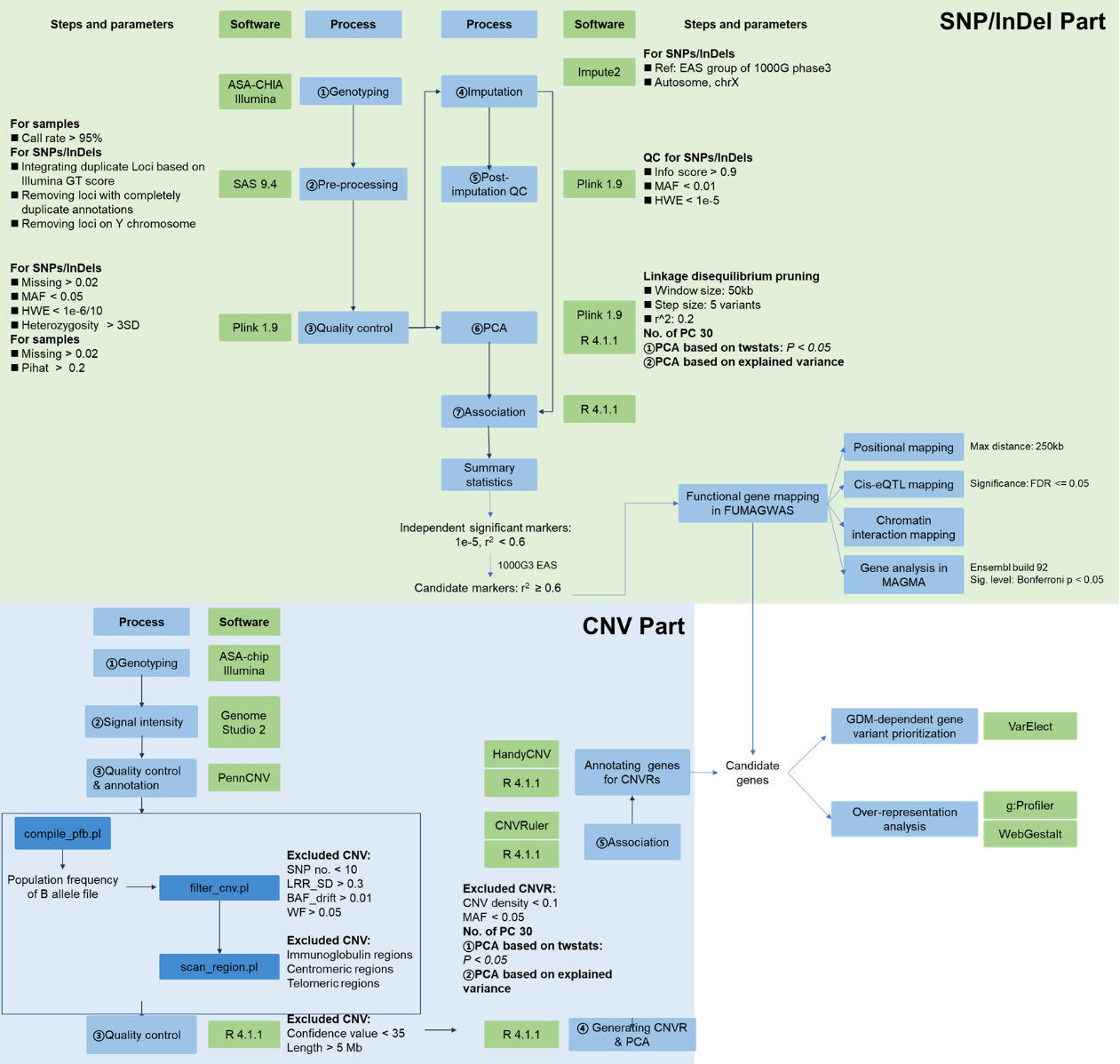


Figure S2. Strategies of the analyses


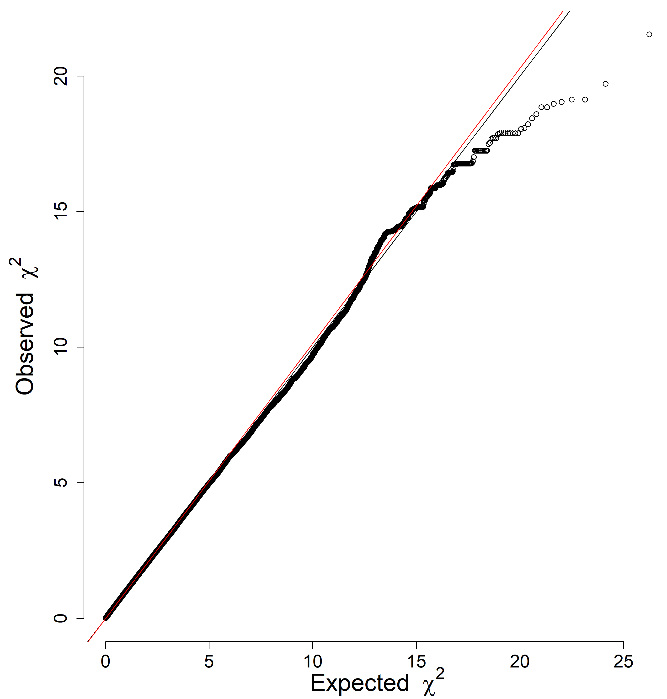


Figure S3. The quantile–quantile plot of P values of SNPs/InDels GWAS (λ=1.01)
